# Intelligence, motoric and psychological outcomes in children from different ART treatments: a systematic review and meta-analysis

**DOI:** 10.1186/s11689-023-09490-0

**Published:** 2023-08-22

**Authors:** Tono Djuwantono, Jenifer Kiem Aviani, Wiryawan Permadi, Danny Halim, Tri Hanggono Achmad, Meita Dhamayanti

**Affiliations:** 1 Department of Obstetrics and Gynecology, Faculty of Medicine, Universitas Padjadjaran/Dr. Hasan Sadikin General Hospital, Bandung, West Java Indonesia; 2 Bandung Fertility Center, Limijati Mother and Child Hospital, Bandung, West Java Indonesia; 3 Research Center for Medical Genetics, Faculty of Medicine, Universitas Padjadjaran, Bandung, West Java Indonesia; 4 Department of Basic Medical Science, Faculty of Medicine, Universitas Padjadjaran, Bandung, West Java Indonesia; 5 Department of Child Health, Faculty of Medicine, Universitas Padjadjaran/Dr. Hasan Sadikin General Hospital, West Java Bandung, Indonesia

**Keywords:** Assisted reproductive treatment, In vitro fertilization, Intracytoplasmic sperm injection, Children neurodevelopment, Intelligence quotient, Motoric skills, Behavioral problems, Toddlers, Preschool and primary school children, Young adolescents

## Abstract

**Background:**

Subtle abnormalities in children’s intelligence, motor skills, and psychology from various assisted reproductive treatments (ARTs) might be underdiagnosed. Understanding the prognosis of intelligence, motor skills, and psychology in children from ART would provide parents with reasonable expectations and enable them to plan relevant support to achieve the optimum potential in ART children.

**Methods:**

We searched PubMed, EMBASE, Ovid, Google Scholar, and Scopus databases until April 13, 2021, to identify relevant studies. Thirty-four studies met the inclusion and exclusion criteria. The meta-analysis employed a standardized mean difference model. The outcome of this study is to compare intelligence quotient (IQ), motoric ability, and behavioral problems between all ARTs, in vitro fertilization (IVF), intracytoplasmic sperm injection (ICSI) to naturally conceived (NC) children. Subdomains of intelligence based on the Cattell, Horn, and Carroll Model (CHC Model) of cognitive architecture, including fluid reasoning, short-term and working memory, processing speed, visual-spatial ability, long-term memory retrieval, and crystalized intelligence (knowledge), were evaluated and summarized in details. Motor skill was stratified into two domains: gross motoric and fine motoric. Behavioral problem was categorized as externalizing and internalizing behavior.

**Results:**

Meta-analysis showed that verbal intelligence score in IVF toddlers is significantly lower than NC toddlers (*p* = 0.02); conversely, ICSI toddlers scored significantly higher verbal intelligence score compared to NC toddlers (*p* = 0.005). Toddlers born after ART had significantly lower non-verbal intelligence score (*p* = 0.047). IVF toddlers scored significantly lower fine motor score (*p* = 0.01) compared to naturally conceived toddlers. Based on parent's CBCL, NC toddlers had higher total (*p* = 0.01) and externalizing behavior (*p* = 0.001) scores  compared to ART toddlers. Evaluation of full scale IQ and all domains of intelligence in preschool and primary school children revealed that no significant differences exist between ART and NC children. Based on preschool and primary school parents' CBCL, IVF children had significantly lower externalizing behavior score compared to NC children (*p* = 0.04). Meta-analyses of studies on young adolescents revealed that ART young adolescents scored higher academically than their NC counterparts, including on mathematics (*p* < 0.00001) and reading or language (*p* < 0.00001).

**Conclusions:**

Despite differences in certain aspects, this finding suggests that ART is unlikely to cause negative impacts on children’s neurodevelopment.

**Supplementary Information:**

The online version contains supplementary material available at 10.1186/s11689-023-09490-0.

## Introduction

Over the past few decades, assisted reproductive technology (ART) has been integrated into the standard protocols to treat infertility. In 2014, there were 1,929,905 ART cycles from 2,746 centers in 76 countries. From 2010 to 2014, the number of reported non-donor aspirations and frozen embryo transfer cycles increased by 37.3% and 67.5%, respectively. The proportion of fresh non-donor single embryo transfers increased from 30.0% in 2010 to 40.0% in 2014 [1].

Since its inception, numerous ART methods have been developed to address a variety of etiologies. Ovulation induction refers to ovarian follicle stimulation by fertility drugs to reverse anovulation or oligoovulation. Gamete intrafallopian transfer (GIFT) involves removing eggs from a woman’s ovaries and placing them in one of the Fallopian tubes along with the man’s sperm. It is used when the fertility problem is caused by sperm dysfunction or idiopathic (unknown cause) infertility. In vitro fertilization is a technique that allows male and female gametes (sperm and egg) to fertilize outside of the female body. This technique is indicated mainly for tubal factor infertility or if the previous methods have failed. Intracytoplasmic sperm injection (ICSI) is a solution to acquire pregnancy(-ies) if most sperms are immotile. The technique involves sperm injection directly into the cytoplasm of a mature oocyte, thus bypassing many natural barriers that prevent natural conception. Despite the superiority of this technique, concerns about preventing defective sperm from fertilizing mature oocytes are frequently raised [2].

Despite the wide use of ART, there are still concerns regarding its safety. How various assisted conception techniques to affect children’s neurodevelopmental outcomes is still unclear. Increased risks of multiple births, preterm birth, and low birth body weight have been described in ART compared to spontaneous pregnancies [3, 4]. Those risks are also associated with neuromotor development disturbances [5]. Our previous meta-analysis showed that children born after ART attain a higher risk for neurodevelopmental disorders, especially cerebral palsy (risk ratio [RR] 1.82, [1.41, 2.34]; *P* = 0.00001) [6]. However, a question regarding subtle clinical manifestations, i.e., intelligence, motor, and mental developments, remains unanswered and less studied. A limited number of studies with various timing of follow-ups, different ART methods, and methodological shortcomings are the major limitations for neurodevelopmental risk interpretation.

There were inconsistent results regarding the neurophysiological and behavioral outcomes of children born after ART. Many of these studies only focused on mental and psychomotor development in the first 3 years of life. Children at preschool to early adolescent ages, when cognitive demand increases, motoric skills are well developed, while socioemotional and behavioral changes are marked, have been insufficiently studied [7].

This study aimed to conclude studies on neurodevelopmental outcomes (intelligence, motoric, and behavior) in children born after different ART treatments compared to naturally conceived (NC) children at every developmental stage: toddlers (1–3 years), preschool to school age (4–8 years) and young adolescents (8–18 years).

## Methods

### Literature search and identification

This meta-analysis was conducted in accordance with the Preferred Reporting Items for Systematic Reviews and Meta-analyses (PRISMA) [8] reporting guidelines. PubMed, EMBASE, Ovid, Google Scholar, and Scopus databases were used to collect publications up to April 13, 2021. The following search terms were applied: (reproductive techniques OR assisted reproductive OR in vitro fertilization) AND (psychomotor performance OR intelligence test OR intelligence quotient OR child behavior OR behavioral test OR temperament).

### Inclusion and exclusion criteria

Studies were included if they (1) reported singleton-born children; (2) reported neurodevelopmental outcome scores on intelligence, language development, motoric skill, socioemotional, or behavior; (3) reported children born from ART techniques; and (4) reported naturally conceived children as control. Studies were excluded if they (1) did not include original data, such as reviews, systematic reviews, comments, or editorial letters; (2) did not include a control group (e.g., case reports); (3) could not ascertain the use of fertility treatment; (4) was not written in English; (5) reported children aged < 12 months; (6) used unstandardized instruments for assessment; (7) reported children born after donor insemination, oocyte donation, or sperm donation; (8) included children with serious health problems or neurodevelopmental disorders.

### Data collection and analysis

Three authors (TD, JKA, DH) reviewed the title and abstract of every article independently. The full-text article was thoroughly read if the abstract met the inclusion criteria. Screening through the reference lists was performed to identify publications that were previously unidentified but relevant to this study. The following information was retrieved: author, country, publication year, number of participants, method of conception, domain, and methods of neurodevelopmental assessment. Newcastle–Ottawa Scale (NOS) was applied to assess the risk of bias in the studies [9].

### Data synthesis

A rigorous review was done by stratifying the result based on age groups, as these groups represent different developmental milestones. In this review, children were grouped into toddler (1–3 years), preschool and primary school age (4–11 years), and young adolescent (12–18 years).

At the age of 1- to 3-year-old, toddlers are advancing their sensorimotor to preoperational intelligence, where they are progressing from learning objects and environment by touch to the development of language and communication. Gross motor skill quickly develops when the transition from crawling to walking and standing occurs. Fine motor skills in this age group are limited to refinements in reaching, grasping, and manipulating small objects. During this period, children are also learning to socialize mainly through playing activity, where they learn cooperation, empathy, and develop friendships with others [10].

Primary school is the first stage of basic education. It bridges early childhood education to formal school education. The programs are typically designed to provide students with fundamental skills in literacy (reading and writing) and mathematics, and to establish a solid foundation for learning. According to ISCED classification, primary education typically starts between the ages of 5- to 8-year-old (1st to 3rd grade). However, in many countries, primary school starts from 4- to 12-year-old (1st to 6th grade). Gross motor is already well developed, and complex fine motoric tasks such as writing and typing can already be performed [11].

At young adolescent ages, children are usually already attending secondary school. Secondary education prepares students for tertiary or higher education and/or provides skills relevant to employment. In this stage, the competencies achieved in primary school are developed in more detail [12]. School grades can be used as a measurement tool of academic intelligence.

By referring to the previously mentioned developmental milestones at different stages of life, three domains of development were assessed: intelligence, motor development, and behavior (social skills). In addition to full scale IQ, two domains of intelligence were assessed: verbal and non-verbal intelligence (Performance IQ). When possible, the subdomains of intelligence based on the Cattell, Horn, and Carroll Model (CHC Model) of Cognitive Architecture, including quantitative intelligence, fluid reasoning, short-term memory and processing speed, visual-spatial ability, long-term memory retrieval, and executive function were evaluated and summarized in details. Verbal intelligence is the ability to understand and reason using concepts framed in words. Verbal IQ is related to crystalized or comprehension knowledgeability in the CHC model. Fluid intelligence is the ability to solve novel reasoning problems and is correlated with essential skills, such as comprehension, problem-solving, and learning. Short-term memory is the capacity for holding a small amount of information in an active, readily available state for a short interval. Processing speed is the ability to perform simple repetitive cognitive tasks quickly and fluently. Visuospatial intelligence is the ability to perceive, analyze, and understand visual information. Long-term memory retrieval is a process of accessing stored memory gained from the learning process [12].

Motoric skills were analyzed in 2 domains: gross motoric and fine motoric. Gross motor (physical) skill is the ability to move the whole body, which involves core stabilizing muscles to perform everyday functions, such as standing, walking, dressing, etc. Fine motor skill is the ability to move minor muscles such as the wrist, hand, fingers, feet, and toes to perform small movements such as picking up objects, gripping, tool manipulation, etc. [13].

Behavioral problems were categorized as externalizing and internalizing behavior. The externalizing spectrum incorporates a variety of disinhibited or externally-focused behavioral symptoms, including aggression, conduct problems, delinquent behavior, oppositionality, hyperactivity, and attention problems. In contrast, the internalizing spectrum includes a variety of over-inhibited or internally-focused symptoms, including anxiety, fear, sadness/depression, social withdrawal, and somatic complaints [14].

Another evaluated aspect was executive function. Executive function is defined as a set of cognitive processes that is necessary for selecting and successfully monitoring behaviors that facilitate the attainment of chosen goals. There are three basic executive function components: inhibition, working memory, and cognitive flexibility. Inhibition is the self-control of attention, behavior, thoughts, and/or emotions to override a strong internal predisposition or external lure and do what is more important. The second aspect is working memory. Working memory is related to the act of holding information (perceptual input) in mind and manipulating or connecting it to bring conceptual knowledge. Working memory is also related to selective, focused attention as the brain will focus on the information held in the mind, turning out irrelevant thoughts. Cognitive flexibility is the third element of executive function. One aspect of cognitive flexibility is being able to change perspective spatially or interpersonally, which is related to inhibition or previous perspective. Higher-order executive functions require the simultaneous use of multiple basic executive functions, including planning and fluid intelligence (e.g., reasoning and problem-solving) [15].

### Statistical analysis

Random effect standardized mean difference (SMD) with a 95% confidence interval was used in the meta-analysis for continuous data. This type of data analysis was used to summarize studies that reported the same outcomes measured in a variety of psychometric scales. Nonetheless, we were aware that this method might be unable to identify real scale differences. RevMan version 5.3 software (Cochrane Collaboration) was used for these purposes. The inconsistency index (*I* ^2^) test, which ranges from 0 to 100%, was performed to evaluate heterogeneity across studies. *P* value < 0.05 or values above 50% indicate a significant heterogeneity. The risk of bias was evaluated by the Cochrane Risk of Bias Assessment tool (Cochrane Collaboration).

## Results

**Fig. 1:**
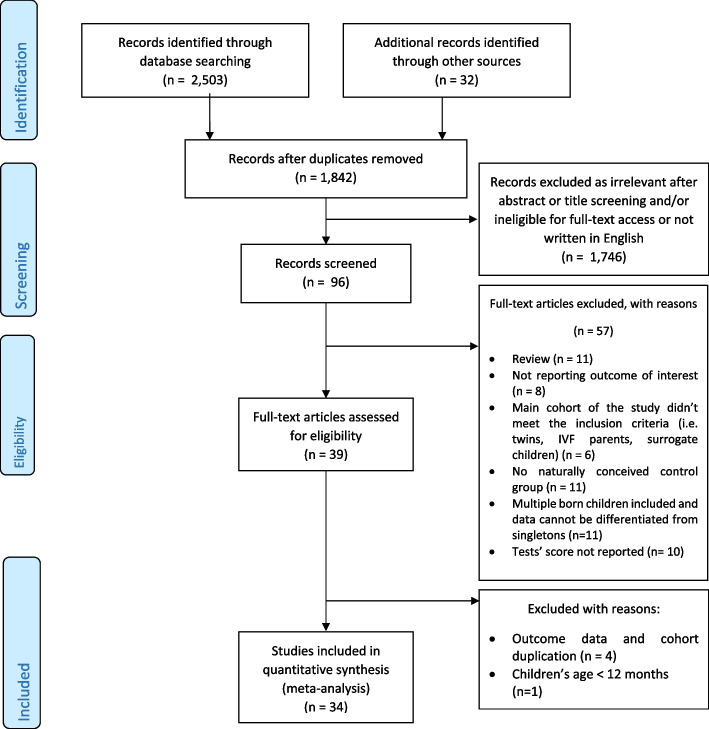
Flow diagram of included study in meta-analysis

The literature searches identified 2503 studies, with the addition of 32 studies identified through reference screening (Fig. 1). Following a review of 96 full-text articles, 57 were excluded for failing to meet the inclusion criteria. Five studies were excluded because they focused on infants under 1 year of age (1 study) and reported duplication of cohort and data in four other studies. Only 34 studies [16–49] were ultimately included in the meta-analyses. The quality of the included studies that were assessed by the Newcastle–Ottawa Scale is shown in Supplemental Table S1 for cohort studies reporting intelligence outcomes, Supplemental Table S2 for case–control studies reporting intelligence outcomes, Supplemental Table S3 for cohort studies reporting motoric outcomes, Supplemental Table S4 for case–control reporting motoric outcome, Supplemental Table S5 for cohort studies reporting behavioral outcomes.

### Characteristic of participants

Table 1 shows pooled analysis of the background characteristics of the children. There were no differences in children’s gender proportion, mother education level, and family socioeconomic background in all age groups born from all types of ART conceptions compared to naturally conceived control.

**Table 1:** Pooled analysis of characteristics of the children from included studies

Characteristics	Age group	Reporting studies	No. of children				Ratio, *p* value	Heterogeneity (*I* ^2^)
			IVF	ICSI	All ART	Control		
Male (%)	Toddler	16, 21, 25		225/497		303/621	0.97 [0.88, 1.07], 0.54	0%, 0.78
		17, 20			129/278	1032/2174	0.93 [0.75, 1.15], 0.52	0%, 0.72
		**Summary**			381/831	1335/2795	0.96 [0.88, 1.05]. 0.41	0%, 0.94
	Preschool	31, 34, 35, 42		122/250		129/250	0.89 [0.63, 1.27], 0.53	0%, 0.91
		34,35	81/164			94/170	0.79 [0.51, 1.21], 0.28	0%, 1.00
		27, 28, 30, 32, 33			156/326	4080/7983	0.89 [0.69, 1.15], 0.38	0%, 0.84
		**Summary**			359/740	4303/8403	0.87 [0.73, 1.05], 0.15	0%, 0.99
	Young adolescent	44–46, 48, 49			6410/12,495	772,649/1,509,646	1.00 [0.98, 1.02], 0.90	0%, 0.78
Mother’s higher education (university or above)	Toddler	16, 21, 25		248/515		296/679	1.04 [0.92, 1.17], 0.58	0%, 0.82
		17, 20			74/154	3868/10,661	1.11 [0.48, 2.55], 0.81	93%, 0.0002
		**Summary**			322/669	4164/11,340	1.07 [0.82, 1.41], 0.60	83%, < 0.00001
	Preschool	31, 34–37, 42		422/779		409/763	0.93 [0.75, 1.16], 0.52	78%, 0.0001
		34, 35	65/164			73/170	0.92 [0.66, 1.29], 0.64	41%, 0.19
		28, 30, 32, 33			139/255	2917/6406	0.93 [0.80, 1.08], 0.33	32%, 0.22
		**Summary**			626/1198	3399/7339	0.94 [0.83, 1.07], 0.37	66%, 0.00005
	Young adolescent	45, 46, 48, 49			2907/11,293	359,623/1,505,567	1.00 [0.81, 1.23], 0.99	87%, < 0.00001
Family financial condition								
Low	Toddler	16, 25		76/480		149/626	0.83 [0.32, 2.13], 0.70	91%, 0.001
		17, 18			53/377	2414/12,662	0.54 [0.13, 2.26], 0.40	90%, 0.001
		**Summary**			129/857	2563/13,288	0.72 [0.41, 1.25], 0.24	85%, 0.0002
	Preschool	34, 35, 38		17/418		15/429	1.17 [0.60, 2.28], 0.64	0%, 0.99
		34, 35	20/173			14/170	1.40 [0.73, 2.69]	0%, 0.99
		**Summary**			37/591	29/599	1.29 [0.81, 2.05], 0.29	0%, 1.00
	Young adolescent	NR	NR	NR	NR	NR	NR	NR
Middle	Toddler	16, 17, 25			344/758	947/2714	1.09 [0.90, 1.31], 0.39	67%, 0.05
	Preschool	34, 35, 38		151/427		166/429	0.97 [0.72, 1.29], 0.89	45%, 0.16
		34, 35	44/164			45/170	1.02 [0.49, 2.12], 0.97	75%, 0.04
		**Summary**			195/591	211/599	0.99 [0.76, 1.29], 0.93	50%, 0.09
	Young adolescent	NR	NR	NR	NR	NR	NR	NR
High	Toddler	16, 17, 25			274/758	1167/2714	1.10 [0.78, 1.54], 0.60	83%, 0.003
	Preschool	34, 35, 38		270/418		276/429	0.95 [0.78, 1.17], 0.64	80%, 0.007
		34, 35	126/173			139/170	0.90 [0.75, 1.08] 0.25	57%, 0.13
		**Summary**			396/591	415/599	0.93 [0.84, 1.04], 0.22	63%, 0.03
	Young adolescent	NR	NR	NR	NR	NR	NR	NR

*NR* Not reported

### Toddler (1- to 3-year-old)

#### Intelligence outcome

**Fig. 2:**
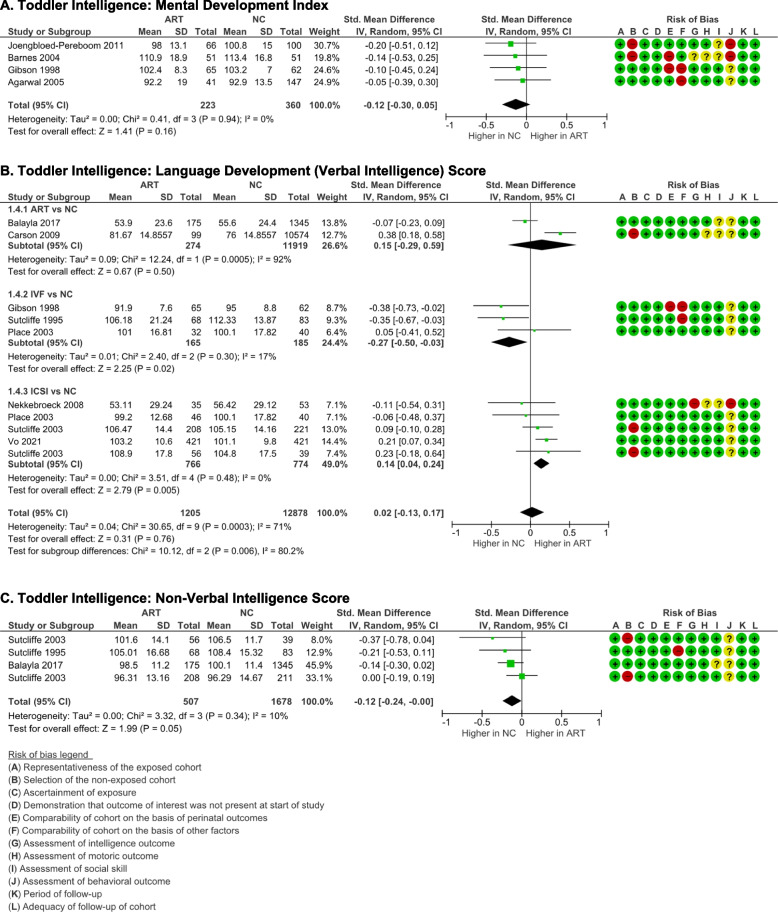
Intelligence outcome of ART-born compared to naturally conceived (NC) toddlers as assessed with **A** Mental Development Index, **B** Language Development (Verbal Intelligence) Score, and **C** Non-verbal Intelligence Score

Four studies used Bayley’s Mental Development Index to measure cognitive development in the toddler age group [16, 19, 20, 26]. There were no significant differences in the mental development of assisted reproductive technology (ART)-born compared to naturally conceived (NC) toddlers (*p* = 0.16). There was no evidence of publication bias (p-Egger = 0.506), and the data exhibited good homogeneity (*I* ^2^ = 0%, *p* = 0.94) (Fig. 2A). Supplemental Table S6 summarizes the statistics for the meta-analysis.

The McArthur Bates Language Inventory [17], British Naming Ability [16], Receptive Expressive Emergent Language-II (REEL-2) [19], McArthur Communicative Developmental Inventories (N-CDI) [21], Brunet-Lezine language sub-scores [22, 25], and Griffith hearing and speech sub-scores [23, 24] were used to measure language development or verbal intelligence. There were no significant differences in language development between ART-born and NC toddlers (*p* = 0.76). Although there was significant heterogeneity (*I* ^2^ = 71%, *p* = 0.0003), the pooled analysis did not indicate publication bias (p-Egger = 0.118) (Fig. 2B). Since the method of conception might affect  heterogeneity, separate subgroup analyses were performed. Good homogeneities were identified in the analyses on IVF vs NC and ICSI vs NC (*p* > 0.05); high heterogeneity was only detected in the analysis on ART vs NC group which included studies that did not specify the mode of conception (*I* ^2^ = 92%, *p* = 0.00005). The language development score of toddlers born after IVF was significantly lower than NC toddlers (*p* = 0.02); meanwhile, ICSI toddlers' score was significantly higher compared to NC toddlers (*p* = 0.005).

Non-verbal intelligence was reported in 3 studies that used Bayley-III cognitive [15] and Griffith performance sub-scores [23, 24]. Pooled analyses showed that non-verbal intelligence in ART toddlers is significantly lower compared to the NC toddlers  (*p* = 0.047) (Fig. 2C). Good homogeneity (*I* ^2^ = 10%, *p* = 0.34) and lack of publication bias (p-Egger = 0.703) were both displayed in these studies.

#### Motoric outcome

**Fig. 3:**
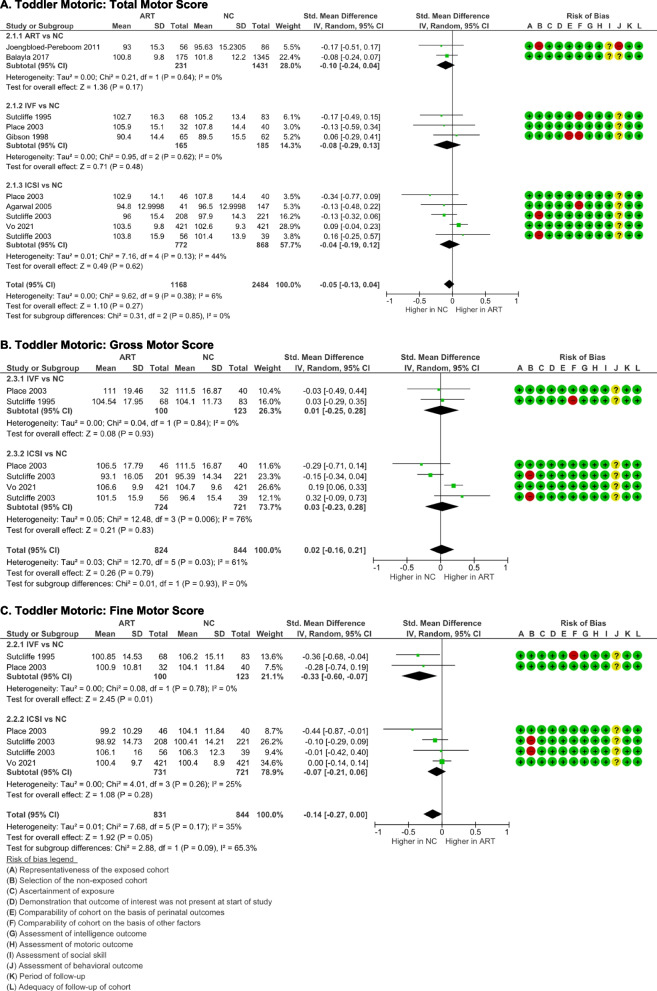
Motoric outcome of ART-born compared to naturally conceived (NC) toddlers as assessed with **A** Total Motor Score, **B** Gross Motor Score, and **C** Fine Motor Score

Bayley-II Psychomotoric Development Index (PDI) [16, 19, 20], Bayley-III motor composite score [15], Brunet-Lezine posture and coordination [22, 25], and Griffith locomotor and eye-hand coordination [22, 24] were utilized to assess the total motor skill outcome. Pooled analysis showed no significant difference in total motor score between toddlers born via ART and naturally conceived toddlers (*p* = 0.27) (Fig. 3A). There were no evidence of data heterogeneity (I^2^ = 6%, *p* = 0.38) and publication bias (p-Egger = 0.575). Similarly, subgroup analyses also revealed the insignificant differences of total motor score in toddlers born from ART, IVF, or ICSI compared to NC toddlers, with good homogeneity and no publication bias (*p* > 0.05).

The gross motor score was obtained from the Griffith locomotor [22, 25] and the Brunet-Lezine posture subtests [23, 24], in both pooled analysis (*p* = 0.79) and subgroup analyses based on the method of conception (IVF, *p* = 0.93; ICSI, *p* = 0.83) (Fig. 3B). Significant heterogeneity between studies was identified (*I* ^2^ = 61%, *p* = 0.03), especially in the ICSI subgroup (*I* ^2^ = 76%, *p* = 0.006), suggesting that factors other than conception mode might also influenced how children developed their motor skills.

The fine motor score in ART and NC toddlers was similar (*p* = 0.055) based on Brunet-Lezine's coordination [, ] and Griffith's ey-hand coordination [, ] assessments. The analyses showed low heterogeneity (*I* ^2^ = 35%, *p* = 0.17) and no publication bias (p-Egger = 0.322). The subgroup analyses revealed that toddlers born after IVF had a noticeably lower fine motor score (*p* = 0.01) than NC toddlers. No significant disparity was noticed in ICSI toddlers compared to NC toddlers (*p* = 0.28) (Fig. 3C).

#### Behavior and social outcomes

**Fig. 4:**
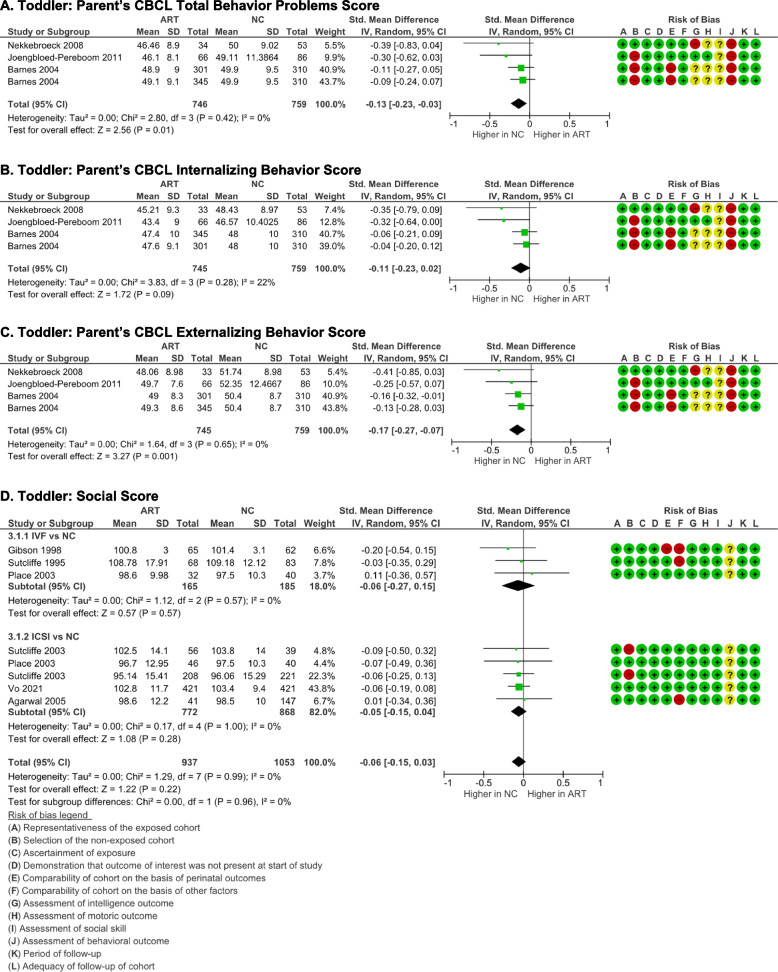
Behavior and social outcomes of ART-born toddlers compared to naturally conceived (NC) toddlers as assessed using **A** Total Behavior Problems Score, **B**  Internalizing Behavior Score, **C** Externalizing Behavior Score, and **D** Social Score

According to three studies, NC mothers reported behavioral issues more frequently than ART mothers, as assessed using Achenbach’s Child Behavioral Checklist (CBCL) [20, 21, 26]. Compared to ART children, NC children showed higher total (*p* = 0.01) and externalizing behavior scores (*p* = 0.001) (Fig. 4A, C). No significant difference was noted in internalizing behavior score between the two groups (*p* = 0.09) (Fig. 4B). The data showed good homogeneities (*I* ^2^ = 0%, *p* > 0.05) and no publication biases.

There was no statistically significant difference observed in the social skills of ART and NC toddlers as assessed using Griffith’s social [23, 24], Brunet-sociability Lezine’s [21, 25], and Vineland Adaptive Behavior socialization [16, 19] (*p* = 0.22) (Fig. 4D). Likewise, there were no significant differences observed in the subgroup analyses between the IVF (*p* = 0.57) and ICSI (*p* = 0.28) toddlers compared to NC toddlers. No heterogeneity (I^2^ = 0%, *p* >0.05), and publication bias (p-Egger > 0.05) were found in the analyses.

### Preschool and primary school ages (4- to 11-year-old)

#### Intelligence outcome

Weschler Preschool and Primary School Intelligence-Revised version (WPPSI-R) [22, 27, 31, 33, 39, 40, 42], Weschler Abbreviated Scale of Intelligence (WASI) [30], Weschler Intelligence Scale for Children (WISC) [36, 37], Kauffman Assessment Battery for Children (K-ABC) [38, 41], and Revised Amsterdam Child Intelligence Test (RAKIT) [35] were used to measure intelligence. There was no significant difference in the overall full-scale IQ of ART schoolers compared to NC schoolers (*p* = 0.31). There was significant heterogeneity observed among the studies (*I* ^2^ = 50%, *p* = 0.01), but no evidence of publication bias was detected (p-Egger = 0.438). ICSI subgroup analysis also demonstrated significant heterogeneity (I^2^ = 59%, *p* = 0.01). Across the three subgroups, the results consistently indicated that there was no significant differencebetween ART and NC schoolers.

**Fig. 5:**
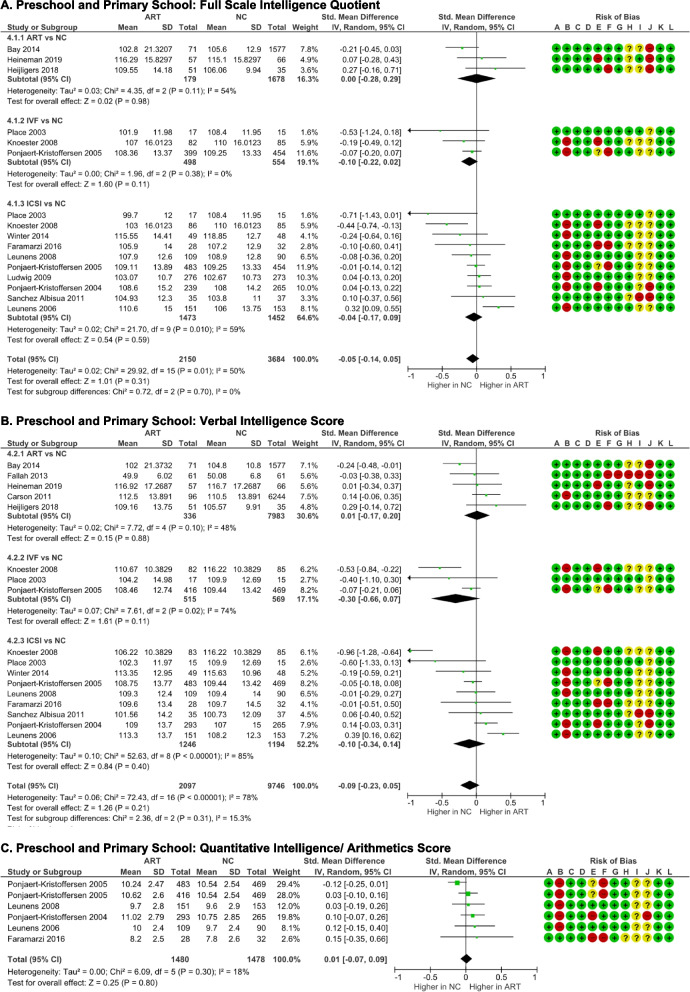

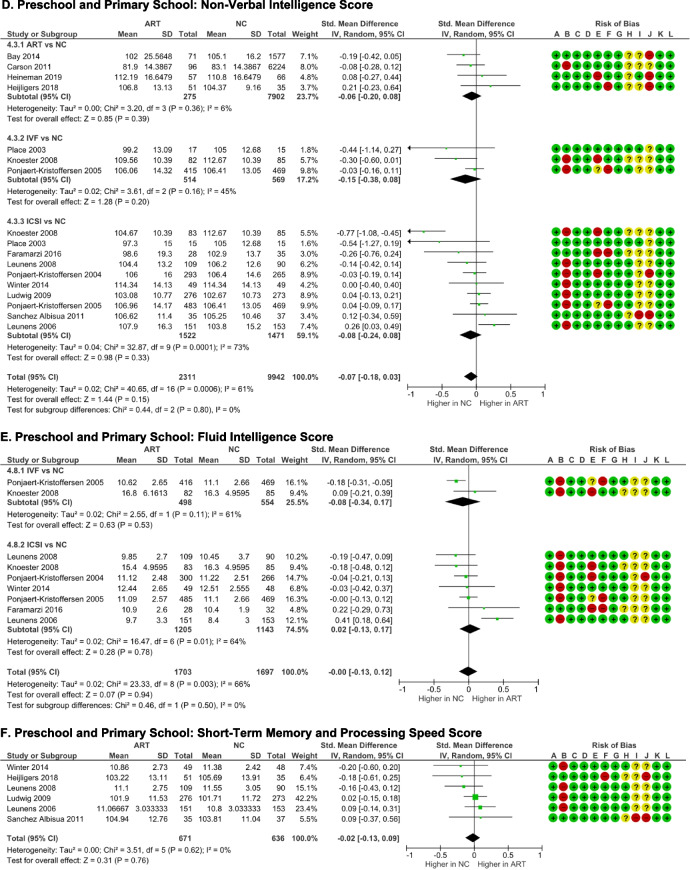

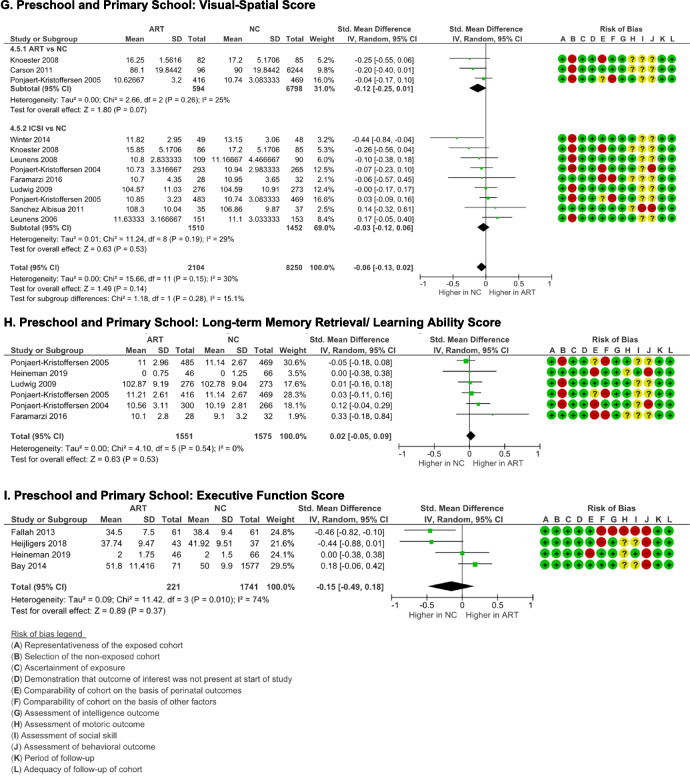
Intelligence outcome of ART-born compared to naturally conceived (NC) pre and primary schoolers  as measured with **A** Full Scale IQ, **B** Verbal IQ, **C** Quantitative Intelligence/Arithmetics, **D** Performance IQ, **E** Fluid Intelligence, **F** Short-term Memory and Processing Speed, and **G** Visual-spatial Intelligence, **H** Long-term Memory Retrieval/Learning Ability **I** Executive Function

The verbal intelligence quotient was calculated from WPPSI-R [22, 27, 31, 33, 39, 40, 42], WASI [32], and WISC [36, 37] verbal IQ, K-ABC Knowledge subtest [41], RAKIT verbal meaning, learning names, and idea production subtests [35], British Ability Scale (BAS) vocabulary subtest [28], and Ages and Stages Questionnaire communication subtest [30] scores. In the subgroup and  overall analyses, no significant differences were found (Fig. 5B). Nonetheless, both the overall (*I* ^2^ = 78%, *p* = 0.0001) and subgroups analyses (*I* ^2^ = 48–85%, *p* < 0.05) demonstrated significant heterogeneity. There were no evidence of publication biases in all groups (p-Egger >0.05).

Quantitative intelligence was extracted from WPPSI, WISC, and WASI arithmetic subtests [31, 36, 37, 39, 40]. There was no significant difference in arithmetic subtest score between ART and NC schoolers (*p* = 0.80) (Fig. 5C). The data exhibited good homogeneity (*I* ^2^ = 18%, *p* = 0.30) and no evidence of publication bias (p-Egger = 0.338).

 Non-verbal intelligence score was obtained from WPPSI-R [22, 27, 31, 33, 39, 40, 42], WASI [32], WISC [36, 37] Performance IQ subtest, K-ABC total score excluding knowledge score [38, 41], RAKIT recognizes figure exclusion, discs, and hidden figures [35], and British Ability Scale (BAS) non-verbal ability [28] scores. According to the total pooled analysis (*p* = 0.15) and subgroup analyses (*p* = 0.20–0.39) (Fig. 5D), ART schoolers had comparable non-verbal score to NC schoolers. Significant heterogeneities were noticed in the pooled (*I* ^2^ = 61%, *p* = 0.0006) and ICSI subgroup (I^2^ = 73%, *p* < 0.0001) analyses. There were no indications of publication biases in all groups (p-Egger > 0.05) (Supplemental Table S6).

Fluid intelligence score was derived from the picture concepts, picture completion, and matrix reasoning subtests of the WPPSI, WISC, and WASI [31, 36, 37, 39, 40, 42], the K-ABC planning subtest [38], and the RAKIT recognize figure exclusion subtest [35] scores. The categorization of CHC intelligence model was based on Keith et al. (2006) [50] for Weschler, Gallagher et al. (2011) [51] for K-ABC, and Jan te Nijenhuis et al. (2004) [52] for RAKIT subtests. As seen in Figure 5E, there were no differences of the fluid intelligence score between IVF (*p* = 0.53) and ICSI (*p* = 0.78) schoolers compared to NC schoolers. The data exhibited moderate heterogeneity (I^2^ = 61-64%); however, no publication biases were observed (p-Egger > 0.05).

Short-term memory and processing speed scores were obtained from the WPPSI, WISC, and WASI picture memory, sequencing, and digit span, coding, and substitution subtests [36, 37], K-ABC sequential processing [38, 41], and automated working memory assessment (AWMA) [33] scores. ART schoolers had comparable short-term memory and processing speed scores with NC schoolers (*p* = 0.76) (Fig. 5F). The data showed homogeneity (*I* ^2^ = 0%, *p* = 0.62) and indicated no publication bias (p-Egger = 0.554).

Visual-spatial intelligence score was determined from the WPPSI, WISC, WASI block design, geometric design, and maze subtests [31, 36, 37, 39, 40, 42], the K-ABC simultaneous processing [38, 41], the RAKIT disks and hidden figures [35], and the British Ability Scale II (BAS-II) spatial ability [28] subtests. There were no discernible differences of visual-spatial intelligence score between ART and NC schoolers, as indicated by total (*p* = 0.14) and subgroups analyses (p ART = 0.07; p ICSI = 0.53) (Fig. 5G). The data exhibited homogeneity and indicated no publication bias.

Long-term memory retrieval/ learning ability score was obtained from WPPSI, WISC, WASI animal pegs, and zoo location [31, 39, 40], K-ABC learning ability [38], and NEPSY domain memory and learning [32] subtests. ART schoolers  exhibited equal learning ability to NC schoolers (*p* = 0.53) (Fig. 5H). The data were homogenous  (*I* ^2^ = 0%, *p* = 0.54), and indicated no publication bias (p-Egger = 0.443).

Executive function score was obtained from the ASQ problem-solving [30], the Behavior Rating Inventory of Executive Function (BRIEF) general executive composite [27, 33], and A Developmental NEuroPSYchological Assessment (NEPSY) domain attention and executive function [32] scores. There was no discernible difference in the executive function score between ART and NC schoolers (*p* = 0.37) (Fig. 5I). Significant heterogeneity was noted (*I* ^2^ = 74%, *p* = 0.010), but there was no evidence of publication bias (p-Egger = 0.533).

### Motoric outcome

**Fig. 6:**
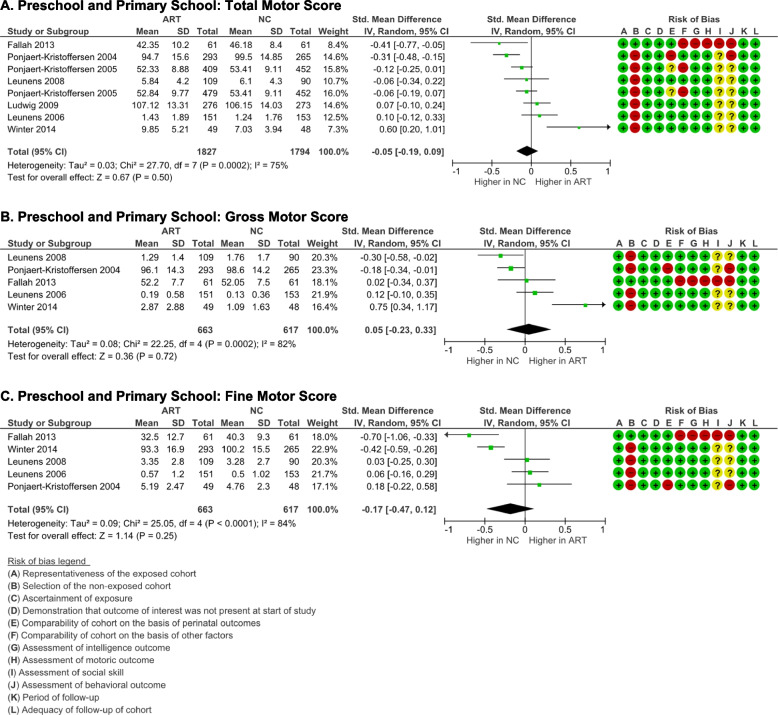
Motoric outcome ART-born compared to naturally conceived (NC) pre and primary schoolers  as assessed using  **A** Total Motor Score, **B** Gross Motor Score, and **C** Fine Motor Score

Total motor score was assessed using the Kauffman ABC Motoric Scale [36, 37, 42], Peabody Development Motor Scale [39], McCarthy Scales of Children’s Ability (MSCA) motor scale index [40], Zimmer/Volkamer Motor Test MOT 4–6 [38], and the ASQ fine and gross motor score [30]. ART schoolers had comparable total motor score with NC schoolers (*p* = 0.50), although high heterogeneity was identified (*I* ^2^ = 75% (*p* = 0.0002) (Fig. 6A), with no evidence of publication bias (p-Egger = 0.399).

Only five studies reported the gross and fine motor sub-scores. The gross motor score was meta-analyzed from Kauffman ABC Motoric Scale ball and balance scores [36, 37, 42], the Peabody Development Motor Scale gross motor quotient [39], and the ASQ gross motor score [30]. The fine motor score was meta-analyzed from K-ABC motoric scale manual score [36, 37, 42], Peabody Development Motor Scale fine motor quotient [39], and ASQ fine motor score [30].  There were no differences in the gross and fine motor scores between ART-born and NC schoolers (*p* = 0.72 and 0.25, respectively). Although there were significant heterogeneities (*I* ^2^ = 82–84%), there were no evidence of publication biases detected (p-Egger > 0.05).

#### Behavior and social outcome

**Fig. 7:**
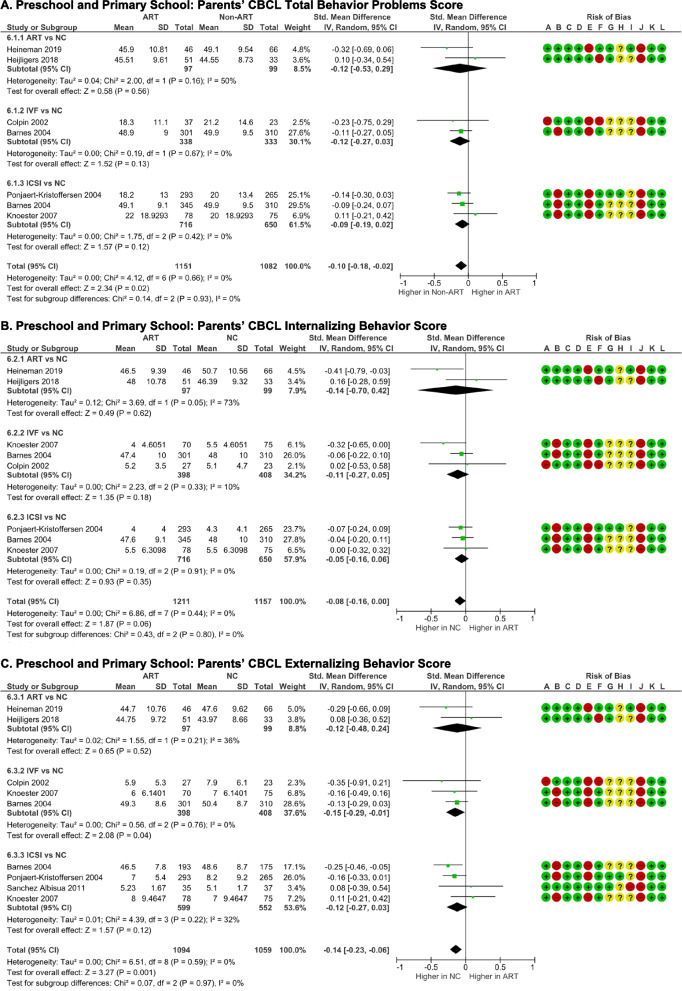

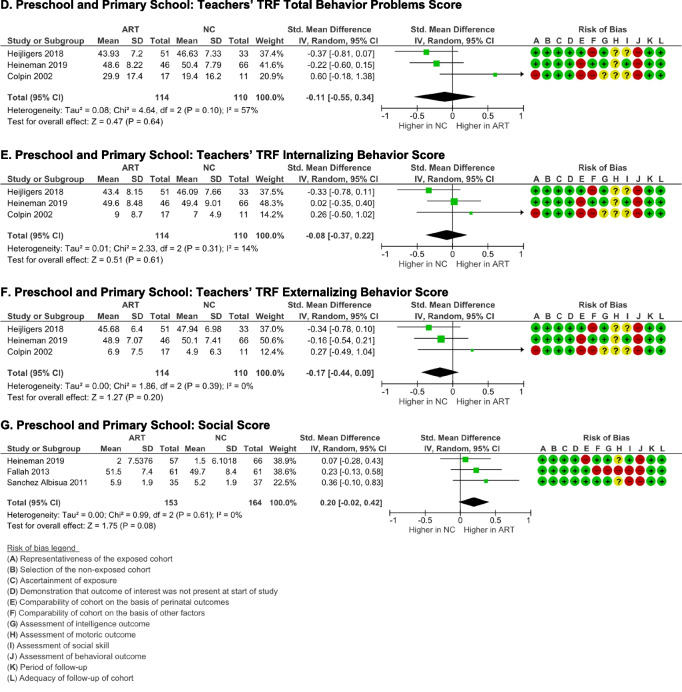
Behavior and social outcomes  of ART-born compared to naturally conceived (NC) pre and primary schoolers assessed using **A** Parents' CBCL Total Behavior Problems Score, **B** Parents' CBCL Internalizing Behavior Score, **C** Parents' CBCL Externalizing Behavior Score, **D** Teachers' TRF Total Behavior Problems Score, **E** Teachers' TRF Internalizing Behavior Score, **F** Teachers' TRF Externalizing Behavior Score, and **G** Social Score

In five studies, preschool and primary schoolers' mothers reported internalizing, externalizing, and total behavioral problems by completing Achenbach’s Child’s Behavior Checklist [22, 29, 32, 33, 39]. Externalizing behavior was also reported in one study using the German behavioral questionnaire for preschoolers, Verhaltensbeurteilungsbogen für Vorschulkinder (VBV), aggressive/oppositional, hyperactivity, and attention subtests [41]. Pooled analysis indicated that NC schoolers exhibited higher total behavior problems score behavioral issues [(*p* = 0.02), *I* ^2^ = 50% (*p* = 0.05)] (Fig. 7A). Internalizing behavior score was not significantly different between the two groups [(*p* = 0.06), *I* ^2^ = 0%, (*p* = 0.44)] (Fig. 7B). However, externalizing behavior score was significantly higher in NC schoolers than ART schoolers [(*p* = 0.001, *I* ^2^ = 0% (*p* = 0.59)] (Fig. 7C).

In three studies, teachers also reported the behavioral problems using the Teacher Report Form (TRF) [29, 32, 33]. Total behavior (*p* = 0.64), internalizing behavior (*p* = 0.61), and externalizing behavior (*p* = 0.20) were not differ  between NC and ART schoolers (Fig. 7D-F). There were moderate data heterogeneities (*I* ^2^ = 0–57%) and no evidence of publication bias (p-Egger > 0.05).

Three studies reported social skills based on the ASQ personal-social [30], NEPSY social cognition domain [32], and VBV social skill subtest [41]. The differences of social scores between ART schoolers  and NC schoolers are insignificant [(*p* = 0.08), *I* ^2^ = 0% (*p* = 0.61)] (Fig. 7D), with no evidence of publication bias (p-Egger = 0.611).

### Young adolescent (12–18 years)

#### Intelligence outcome

**Fig. 8:**
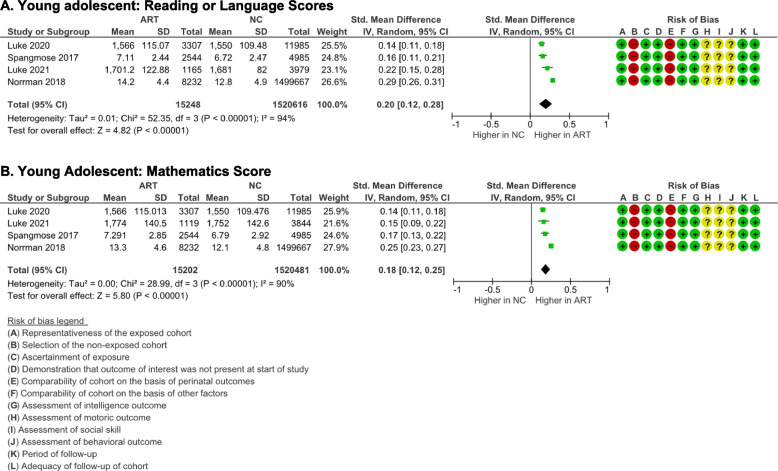
Intelligence outcome of ART-born compared to naturally conceived (NC) young adolescents as assessed using **A** Reading/Language Score and **B** Mathematics Score

Intelligence in the young adolescent age group was measured from school subject’s test scores [43–46]. ART students scored significantly higher than NC students in reading or language (only from native language score) (*p* = 0.00001), although significant heterogeneity was acknowledged (*I* ^2^ = 94%, *p* = 0.00001) (Fig. 8A). Similarly, meta-analysis also revealed that ART students scored significantly higher in mathematics (*p* = 0.00001), although significant heterogeneity was also identified (*I* ^2^ = 90% (*p* = 0.0001) (Fig. 8B). Publication bias was detected in the analysis on mathematics score (p-Egger = 0.025), but not in the analysis on language score (p-Egger = 0.104).

#### Behavioral outcome

**Fig. 9:**
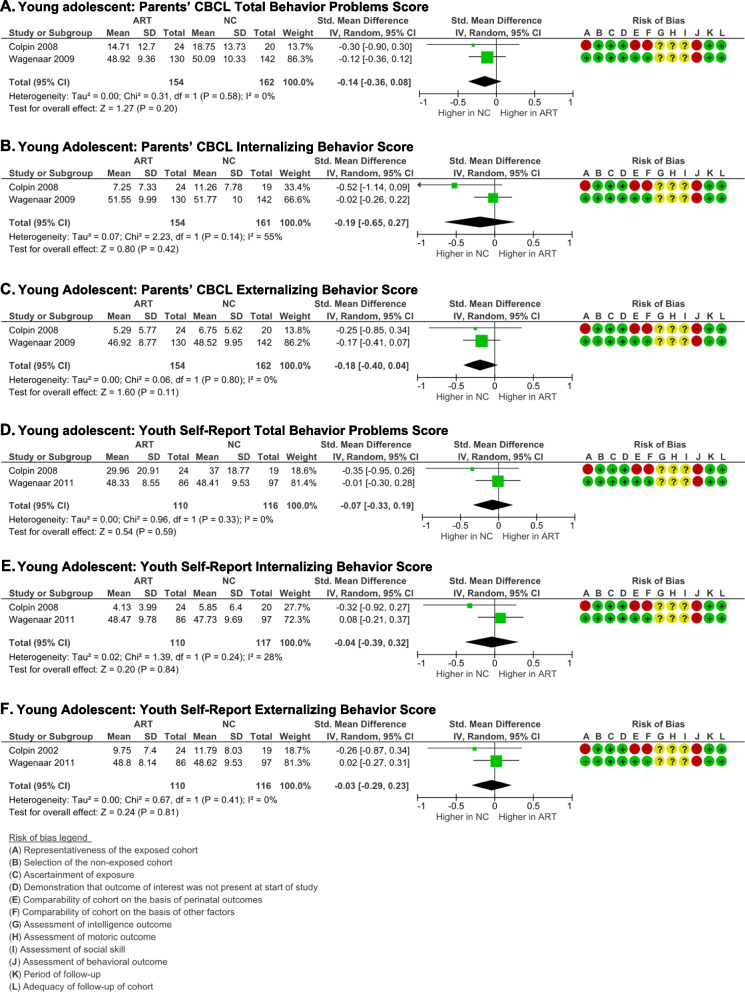
Behavior outcome of ART-born compared to naturally conceived (NC) young adolescents as assessed using **A** Parents' CBCL Total Behavior Problems Score, **B** Parents' CBCL Internalizing Behavior Score, **C** Parents' CBCL externalizing Behavior Score, **D** Youth Self-report Total Behavior Problems Score, **E** Youth Self-report Internalizing Behavior Score, and **F** Youth Self--report Externalizing Behavior Score

The Achenbach Children Behavior Checklist completed by parents  and the Achenbach Youth Self-Report were used to measure behavioral outcomes in the young adolescent group [47–49]. No significant differences between ART and NC young adolescents were identified on total behavioral problems [(*p* = 0.20), *I* ^2^ = 0% (*p* = 0.58)] (Fig. 9A) and (*p* = 0.59), *I* ^2^ = 0% (*p* = 0.33) (Fig. 9D)], internalizing behavior [(*p* = 0.42), *I* ^2^ = 55% (*p* = 0.14)] (Fig. 9B) and (*p* = 0.84), *I* ^2^ = 28% (*p* = 0.24)] (Fig. 9E), and externalizing behavior [(*p* = 0.11), *I* ^2^ = 0% (*p* = 0.80) (Fig. 9C) and (*p* = 0.81), *I* ^2^ = 0% (*p* = 0.41) (Fig. 9F)], as reported by parents and the young adolescents themselves respectively.

#### Obstetrics and neonatal characteristics

Table 2. shows obstetrics and neonatal characteristics in ART and NC groups. According to the data from all age groups, babies born after ART typically have lower gestational ages. They also had a 1.58 to 2.34 times higher risk of preterm birth (gestational age < 37 weeks) and 2.44 to 4.48 times higher risk of low birth weight (birth weight < 2500 g).

**Table 2:** Pooled analysis of obstetric and neonatal characteristics

**Characteristics**	**Age Group**	**Reporting studies**	**No. of Children** **(cases / total)**			**Effect Size, ** *p* **-value**	**Heterogeneity** **(I** ^**2**^ **), ** *p*-value
			**ICSI**	**All ART**	**Control**		
Gestational Age [mean + st. dev (no. of children)]	Toddler	14, 15, 19, 21		38.33 + 2.19 (422)	38.98 + 1.88 (2,371)	-0.57 [-0.81, -0.34], <**0.0001**	34%, 0.21
	Pre-school	35 – 39, 42	39.56 + 1.47 (971)		39.61 + 1.55 (915)	-0.04 [-0.19, 0.10], 0.55	0%, 0.58
		27, 32, 33		39.79 + 1.32	40.19 + 1.39	-0.40 [-0.64, -0.15], **0.002**	0%, 0.74
		**Summary**		39.62 + 1.43 (1,139)	39.75 + 1.51 (2,539)	-0.13 [-0.25, -0.01]**, 0.04**	22%, 0.25
	Young Adolescent	48, 49		38.93 + 2.48 (225)	39.60 + 1.80 (240)	-0.67 [-1.07, -0.28], **0.00009**	0%, 0.85
Preterm birth (Gestational age < 37 weeks)	Toddler	14, 23	10/76		13/200	2.11 [0.95, 4.66], **0.04**	0%, 0.94
		16, 18		26/165	695/10,661	2.23 [1.02, 4.87], **0.04**	57%, 0.13
		**Summary**		36/341	708/ 10,861	2.34 [1.65, 3.33], **<0.00001**	0%, 0.46
	Pre-school	31,32, 34-37, 39, 42		62/852	38/825	1.58 [1.07, 2.32], **0.02**	57%, 0.02
	Young Adolescent	44 – 46, 48, 49		1,236/ 12,484	81,341/ 1,507,453	1.90 [1.80, 2.01], **<0.00001**	92%, <0.00001
Birthweight [mean + st. dev (no. of children)]	Toddler	14, 19	3,073.65 + 608.15 (76)		3,129.3 + 554.40 (200)	-55.65 [-220.91, 109.60], 0.51	0%, 0.96
		15, 18, 21		3,304.89 + 655.84 (412)	3,455.56 + 601.44 (2,258)	-150.48 [-275.36, -25.60], **0.02**	43%, 0.17
		**Summary**		3,255.96 + 640. 54 (488)	3,359.01 + 557.51 (2,458)	-103.00 [-167.48, -38.53], **0.002**	0%, 0.42
	Pre-school	31, 35 – 39, 42	2,791.99 + 558.17 (999)		3,414.624 + 529.12 (947)	-621.97 [-672.43, -571.51], **<0.00001**	100%, <0.00001
		27, 32, 33, 35		3,358.63 + 595.67 (251)	3,572.94 + 531.94 (1,763)	-213.61 [-303.09, -124.14]**<0.00001**	0%, 0.93
		**Summary**		2,911.08 + 558.76 (1,167)	3,447.96 + 525.37 (2,625)	-538.10 [-583.05, -493.14], **<0.00001**	100%, <0.00001
	Young Adolescent	48, 49		3,254.18 + 633.29 (225)	3,413.05 + 476.93 (240)	-158.89 [-261.37, -56.41], **0.002**	0%, 0.45
Low Birthweight (Birthweight < 2,500g)	Toddler	14, 16	20/140		572/ 10,721	2.44 [1.57, 3.79], **<0.0001**	0%, 0.56
	Pre-school	30, 32, 34, 35		29/275	7/297	4.48 [1.99, 10.09], **0.0003**	0%, 0.90
	Young Adolescent	45, 46, 48, 49		956/ 11,349	31,653 / 1,502,580	3.40 [3.18, 3.64], **<0.00001**	95%, <0.00001

## Discussion

This meta-analysis acknowledged that verbal IQ is significantly lower in IVF toddlers, but higher in ICSI toddlers, compared to NC toddlers. Furthermore, non-verbal intelligence is significantly lower in ART compared to NC toddlers. There are no discernible differences in all areas of intelligence between ART and NC preschool and primary schoolers. Interestingly, meta-analyses showed that ART young adolescents had higher intelligence scores compared to NC young adolescents. Fine motor score in IVF toddlers is significantly lower; nonetheless, there were no differences in the ICSI group or total group analysis compared to NC toddlers. In preschool and primary school groups, no differences were found in total motor, gross motor, and fine motor scores between ART and NC children.

We hypothesize that there are several factors that might affect these outcomes. First, in the toddler group, IVF conception was only reported in 3 studies [19, 22, 23], and 2 of them [19, 23] were reported in 1995 and 1998, respectively. We speculate that changes in protocols in IVF might play roles in determining the children’s development. For example, before 2001, there was no preimplantation genetic screening. Improvements in IVF, freeze-thawing, and oocyte retrieval methods have resulted in higher pregnancy and assured higher quality of implanted embryos [53].

While non-verbal intelligence involves parietal lobes and is linked to white matter microstructure, verbal intelligence is related to cortical structure and thickness of the temporal lobes and temporal pole lateral areas. Lower white matter tract integrity has a significant negative impact on general intelligence [54]. The lateral rostral medulla region of the brain stem controls fine motor function [55]. Recent research discovered that single nucleotide polymorphisms have functional effects on neurogenesis, neuronal differentiation, or the structure or activity of synapses [54]. To avoid any genetic defects, the quality of the transferred embryo is crucial in the ART procedure.

However, a study by Zhang et al. [56] revealed that singleton children born following a poor-quality embryo transfer had comparable full-scale, verbal, and performance intelligence as measured with the Weschler Preschool and Primary Scale of Intelligence in comparison to children born following a good-quality transfer. Thus, other factors might have a more significant role in intelligence and motoric ability development.

Second, as shown in Table 2., prematurity and low birth weight were inexplicably more common in ART children. According to a study by Nagy et al. [57], children who were born preterm and those who were underweight at birth performed worse on tests of intelligence and executive function than children who were born full-term, although their results were still within the normal range on average [57]. According to Casey et al., low cortical volume and surface area are related to low birth weight [58]. Advanced imaging techniques revealed that the sensory-motor pathway matured more quickly in preterm infants; however, areas of injury and disturbed development are also visible in their parietal white matter. 

The corpus callosum left inferior longitudinal fasciculus, and left dorsal visual stream mature more slowly in preterm infants. However, if the infant is healthy, these areas will eventually develop more quickly [59].

Lastly, external factors might also contribute, especially to children’s intelligence. According to the findings, the development of ART children at later stages of life is arguably superior to that of NC children. Since this study only included singletons, ART children were probably the first to be born and may have had fewer siblings. Additionally, the likelihood of their parents cohabiting, remaining married, being employed, and having higher socioeconomic, occupational, and educational levels [43–46] helped to improve early cognitive stimulation, which impacts academic performance.

According to parental reports, NC children in the toddler, preschool, and primary school age groups had more behavioral issues.

In contrast, according to their teachers, there were no discernible differences. There were no differences between the young adolescent group’s self-reports and those of their parents. As it solely depends on parents’ perceptions regarding the question related to their children’s behavior, this self-reporting questionnaire method may introduce potential methodological bias.

Lower birth weight, which is more common in ART children, had a significant impact on limbic network connectivity, which is in charge of emotion regulation and internally generated thoughts [60]. However, since all of the children in these studies had scores within normal ranges, we surmise that their limbic development was normal based on the most recent results. The influence of parenting factors on a child’s externalizing and internalizing behavior may be more significant. Parenting stress impact externalizing behavior, whereas parenting negative engagement impact internalizing behavior [61]. Compared to naturally fertile mothers, ART mothers express more warmth and positive feelings toward their children and greater parental competence [26, 62, 63]. These results may indicate a tendency to report socially acceptable responses, given that those behavior problems were assessed using a self-reported questionnaire [64].

### Limitations

The evidence is arguably weak because the current study is a systematic review based on a limited number of studies. A type II statistical error or false negative may result from a small sample size. This occurs when the null hypothesis—which claims no differences between the two groups being compared—is incorrect but still accepted [65]. The second drawback stems from the fact that the analyzed studies used a variety of instruments with various scales, resulting in the evaluation of distinct areas of motoric and intellectual development. This restriction may have introduced bias due to heterogeneity.

Subtest categorization and standardized mean differences based on tested theory can overcome this drawback. Third, the widely used method for evaluating children’s behavioral issues is based on self-reports, which may have information bias. Fourth, the included studies did not mention any additional pediatric medical conditions that might impact the results of their neurodevelopmental studies. For instance, none of the studies mentioned bronchopulmonary dysplasia, a condition frequently associated with brain abnormalities in very preterm infants [66].

## Conclusion

This meta-analysis identified differences on certain aspects of intelligence between ART and NC children. The non-verbal intelligence score of ART toddlers was significantly lower than that of NC toddlers; however, preschool and primary school ART children showed comparable results in all areas of intelligence compared to their NC counterparts. Interestingly, ART young adolescents scored significantly higher academic scores than NC young adolescents. ART toddlers had significantly lower fine motor skills. Parents of naturally born toddlers and school-age children reported more overall behavioral problems. However, behavior scores of young adolescents from both groups were comparable. These results may be influenced by both internal and external variables, including the year of ART procedures, prevalence of prematurity and low birth weight, family socioeconomic background, and parenting style.

### Supplementary Information

**Additional file 1: Supplement Table 1.** Newcastle-Ottawa Scale for Cohort Studies: Intelligence, School Performance, Language Development. **Supplement Table 2.** Newcastle-Ottawa Scale for Cohort Studies: Motoric Development. **Supplement Table 3.** Newcastle-Ottawa Scale of Cohort Studies: Behavioral and Social Development. **Supplement Table 4.** Newcastle Ottawa Scale for the Case-Control Studies. **Supplement Table 5.** Characteristic of the Included Studies. **Supplement Table 6.** Summary of Meta-analysis.

## Data Availability

All data generated or analyzed during this study are included in this published article [and its supplementary information files].

## Abbreviations

ART: Assisted reproductive treatment
ASQ: Ages and Stages Questionnaire
BAS: British Ability Scale
BRIEF: Behavior Rating Inventory of Executive Function
CBCL: Child’s Behaviour Check List
CHC: Cattell, Horn, and Carroll
ICSI: Intracytoplasmic sperm injection
IQ: Intelligence quotient
IVF: In vitro fertilization
K-ABC: Kauffman’s Assessment Battery for Children
MDI: Mental Development Index
MSCA: McCarthy Scales of Childrens Ability
NC: Naturally conceived
NEPSY: A Developmental NEuroPSYchological Assessment
PDI: Psychomotor Development Index
PIQ: Performance intelligence quotient
RAKIT: Revised Amsterdam Child Intelligence Test
REEL: Receptive Expressive Emergent Language
SMD: Standardized mean difference
VBV: Verhaltensbeurteilungsbogen für Vorschulkinder
VIQ: Verbal intelligence quotient
WASI: Weschler Abbreviated Scale of Intelligence
WISC: Weschler Intelligence Scale for Children
WPPSI: Weschler Preschool and Primary Scale of Intelligence
