# Supplementary material for: Intelligence, motoric and psychological outcomes in children from different ART treatments: a systematic review and meta-analysis
Source: J Neurodev Disord. 2023 Aug 22;15:26. doi: 10.1186/s11689-023-09490-0 (PMC10463915; doi:10.1186/s11689-023-09490-0)
Supplement: Supplementary file 1 — Additional file 1: Supplement Table 1. Newcastle-Ottawa Scale for Cohort Studies: Intelligence, School Performance, Language Development. Supplement Table 2. Newcastle-Ottawa Scale for Cohort Studies: Motoric Development. Supplement Table 3. Newcastle-Ottawa Scale of Cohort Studies: Behavioral and Social Development. Supplement Table 4. Newcastle Ottawa Scale for the Case-Control Studies. Supplement Table 5. Characteristic of the Included Studies. Supplement Table 6. Summary of Meta-analysis. [file 11689_2023_9490_MOESM1_ESM.docx]

**Supplement Table 1.** Newcastle-Ottawa Scale for Cohort Studies: Intelligence, School Performance, Language Development

| **Name of Study (Year)** | **Selection** | | | | **Comparability** | **Outcome** | | | **Additional** | **Total Score** |
| --- | --- | --- | --- | --- | --- | --- | --- | --- | --- | --- |
|  | Representativeness of the exposed cohort  (a) truly representative of the average IVF treated subjects in the community*  (b) somewhat representative for the IVF treated subjects in the community*  (c) selected group of users  (d) no description of the derivation of the cohort | Selection of the non-exposed cohort  (a) drawn from the same community as the exposed cohort*  (b) drawn from different source  (c) no description of the derivation of the non-exposed cohort | Ascertainment of exposure  (a) secure record*  (b) structured interview*  c) written self report  (d) no description | Demonstration that outcome of interest was not present at start of study  (a) yes*  (b) no | Comparability of cohort on the basis of the design or analysis  (a) study controls for family background (education, SES, etc.)*  (b) study controls for any additional factors* | Assessment of outcome  (a) Independent blind assessment*  (b) Record linkage*  (c) Self-report  (d) No description | Was follow-up long enough for outcomes to occur  (a) yes (1-year minium follow-up)* (b) no | Adequacy of follow-up of cohort  (a) complete follow-up, all subjects accounted for*  (b) subjects lost to follow-up are unlikely to introduce bias – small number lost <20%  (c) follow-up rate <80% and no description of those lost  (d) no description *or unclear* | Is mode of conception and embryo transfer method stated?  (a) Mode of conception (ICSI, IVF, etc.) stated*  (b) ET method (fresh/frozen stated)*  (c) Twins vs singletons?*  (d) No description |  |
| [Agarwal et al. (2005)](about:blankAgarwal%20et%20al.%20(2005)) | (c) Selected groups from Kandang Kerbau Hospital Singapore | (a) The control group comprised of naturally conceived children born during the same period at the same hospital with matching criteria* | (a) Secure record from hospital records* | (a) Neuro - developmental delay was not present at time of birth* | (a,b) Family background and other confounders are adjusted for in the statistical analysis** | (a) Inependent blind by medical diagnosis, interview, and Bayley Scale for Infant Development, Vineland score* | (a) Yes, Follow-up was done at 2 years of age* | (b) Eighty-five ICSI and 269 naturally conceived children were eligible to be enrolled in the study. Of these, parents of nine study (10%) and eight control children (3%) declined to participate in the study. Thus, 76 ICSI children and 261 controls were assessed at two years of age* | (a) ICSI, NC*  (c) Twins and singletons* | 8 (2) |
| [Balayla et al. (2017)](about:blankBalayla%20et%20al.%20(2017)) | (b) Data from the 3D-study (Decouvrir, developper, Devenir) that recruited women in first trimester in 9 sites in Quebec, Canada* | (a) Non-ART children data from 3D study with matching criteria* | (a) Secure record* | (a) Neurological outcomes were not tested at time of birth* | (a,b) Adjusted for maternal age, paternal age, maternal education, income, ethnicity, marital status, smoking intake, alcohol consumption during pregnancy, history of depression, antidepressant use, and folic acid intake** | (a) Independent medical diagnosis Bayley Scales of Infants and Toddlers at 2 years of age, McArthur Bates for language development* | (a) Yes, follow up until 2 years of age* | (c)  175 children in ART (62.9%)  1345 – NC group (64.4%)  Underwent neurodevelopmental assessment at 24 months postpartum* | (b) Singleton only  Fertility treatment: IVF, ICSI, etc. | 8 (1) |
| [Bay et al. (2014)](about:blankBay%20et%20al.%20(2014)) | (a) A cohort of 1782 children sampled from the Danish National Birth Cohort* | (a) Children born from fertile parents from the same population with matching criteria* | (b) Structrued interview* | (a) Yes, intelligence were not assessed at time of birth* | (a,b) Adjusted for maternal age, intelligence, parental educational level, parity, smoking in pregnancy, average alcohol consumption in pregnancy, maternal body mass index, child gender, age at testing, and testing psychologist** | (a) Independent assessment with WPPSI-R, Teach, BRIEF* | (a) Yes, follow up were done at 5 years of age* | (c) Overall number of participants. Due to complete case analyses, the number of participants for the individually outcomes was: Full IQ (n=1729), Verbal IQ (n=1730), Performance IQ (n=1730), Overall attention score (n=1477), Sustained attention score (n=1570), Selective attention score (n=1595), General Executive Composite (parent version:n=1727, teacher version:n=1507), Behavioural Regulation Index (parent version: n=1727, teacher version:n=1512), Metacognition index (parent version:n=1727, teacher version:n=1507) | (b) Singleton only | 8(1) |
| Bowen et al. (1998)a | (c) Children born at North Shore Hospital, Sydney, Australia | (a) Naturally conceived children born from the same hospital with matching criteria* | (a) Hospital secure record* | (a) Cognitive and motoric assessment were not tested at time of birth* | (a,b) Inclusion of demographic variables as covariates in analysis of variance** | (a) Independent assessment using Bayley Scale II* | (a) Yes, follow up were done at 1 year of age | (a) Complete data during assessment* | (a) IVF vs ICSI vs NC*  Singletons and twins no subgroups  Fresh and frozen no subgroups | 7(1) |
| [Gibson et al. (1998)](about:blankGibson%20et%20al.%20(1998)) | (c) Children conceived at Royal North Shore Hospital IVF Clinic | (a) Children conceived from the same hospital with same inclussion criteria* | (a) Secure hospital record* | (a) Intelligence disturbance were not seen at time of birth* | (a,b) Family background and maternal age were matched in the experimental design** | (a) Independent assessement with  + Bayley scales of infant development  + Receptive-Expressive Emergent Language Test (REEL-2)  + Vineland Adaptive Behaviour Scales  + Bayley Behavior Rating Scale  + Short Temperament Scale for Toddlers (STST)* | (a) Yes, infants were assessed at 1 year post partum* | (b) IVF 93% (n=65)Control 98% (n=62)* | (c) Singleton only* | 8 (1) |
| Goldbeck et al. (2009)a | (c) children conceived at 2 clinics in Ulm, Germany | (c) No controls of naturally conceived children | (a) Secure hospital record* | (a)Yes, children cognitive level were not assessed at time of birth* | (a,b) Family background and other confounders are not matched in the design , however No significant differences appeared between ICSI singletons and IVF singletons with respect to socio-demographic, socio-economic, and medical variables except the method of fertilization** | (a) Independent assessment with Kaufmann Assessment Battery for Children* | (a) Yes, children was at 5 and 10 years of age* | (a) Complete follow-up* | (a) IVF vs ICSI*  (c) Singleton only* | 7 (2) |
| [Heineman et al. (2019)](about:blankHeineman%20et%20al.%20(2019)) | (c) Children from the Groningen ART study at Department of Reproductive Medicine of the UMCG | (a) Naturally conceived children with matching criteria* | (a) Secure hospital record* | (a) Yes, children cognitive level were not assessed at time of birth* | (a,b) There were no significant differences in all background characteristics (Table I) between the children who had and who did not have follow-up at the age of 9 years** | (a) Independent assessment with WASI for intelligence, CBCL and TRF for behavioral score, and NEPSY for executive function and attention* | (a) Yes, children was at 9 years of age* | (a) No missing data* | (a) IVF and ICSI*  (b) Singletons*  (c) Fresh only* | 8(3) |
| [Jongbloed-Pereboom et al. (2011)](about:blankJongbloed-Pereboom%20et%20al.%20(2011)) | (c) Children conceived at University Medical Center Groningen, Netherlands | (a) Naturally conceived children from the different clinics* | (a) Secure hospital record* | (a) No intelligence disturbance were seen at time of enrollment* | (a,b) Mean differences adjusted for maternal education, sex, gestational age, vanishing twins, birth weight, and time to pregnancy** | (a) Independent assessment with  Bayley scales of infant development II  + Achenbach Child Behavior Checklist (CBCL)* | (a) yes, children were at 2 years of age at time of study* | (a) Only 1 missing data from PDI scale* | (a) IVF ICSI*  (b) Singletons*  (c) Fresh only* | 8(3) |
| Knoester et al. (2008)a | (c) Children conceived at University medical center, Leiden, Netherlands | (a) Naturally conceived children drawn from the surrounding community with matching criteria* | (a) Secure hospital record* | (a) No intelligence disturbance were seen enrollment (birth)* | (a,b) Adjusted for maternal education, parity, SES, birthweight, gestational age** | (a) Indepdendent assessment with Revised Amsterdam Child Intelligence Test (RAKIT)* | (a) yes, children was at 5 to 10 years at assessment* | (a) Complete follow up* | (a) ICSI, IVF, NC*  (b) Fresh only*  (c) Singleton only* | 8 (3) |
| Kuiper et al. (2017)a | (c) Children born in Amsterdam Medical Center and University medical center Groningen | (c) No description of naturally conceived children | (a) Hospital secure record* | (a) ASD and other neurological outcomes were not reported at time of enrollment (birth)* | (a,b) family background and other confounders are not matched in the design, however statistical analysis shown no differences in the family backgrounds and other cofounders** | (a) Independent blind assessment with K-ABC, Hempel Assessment* | (a) Yes, follow up until 4 years of age* | (b) Missing data in the two groups: Apgar score 5 min (n= 2); gestational diabetes (n= 1); high education level father (n= 5); maternal BMI at infant conception (n= 9); paternal age at infant conception (n= 21); pregnancy-induced hypertension (n= 1); smoking mother during pregnancy (n= 1)* | (a) IVF-ICSI with ovarian stimulation*  (b) Singleton and twins*  (c) Fresh only* | 8 (3) |
| Leslie et al. (2003)a | (c) Children conceived by IVF and ICSI in North Shore hospital, Sydney, Australia | (a) Naturally conceived children were drawn from local schools with matching criteria* | (a) Hospital secure record* | (a) Intelligence quotient were not assessed at time of enrollment (birth)* | (a,b) Family backgrounds, preterm birth, twins were provided for statistical analysis** | (a) Independent blind assessment with Wechsler Preschool and Primary Scales of intelligence* | (a) yes, follow up for 1 and 5 years of age* | (b)  **Unavailable**  16/89 ICSI  4/84 IVF  20/80 NC* | (a) IVF, ICSI, NC*  No distiction in twins and singleton data | 8 (1) |
| [Leunens et al. (2006)](about:blankLeunens%20et%20al.%20(2006)) | (c) children conceived in Academic Hospital of Vrije Universiteit Brussel, Belgium | (a) Naturally conceived children from local schools with matching criteria* | (a) Hospital secure record* | (a) No intelligence disturbance were seen at time of enrollment (birth)* | (a,b) Family background, gestational age, birthweight were not significantly different in statistical analysis** | (a) Independent blind assessment with  Wechsler Intelligence Scale  + Movement Assessment Battery for children  + Questionnaire* | (a) yes, follow up were done for 8 year old children* | (d) No description | (a) ICSI vs NC*  (b) Singleton only* | 7(2) |
| [Leunens et al. (2008)](about:blankLeunens%20et%20al.%20(2008)) | (c) Children from previous study at age 10 years | (a) Naturally conceived children from local schools with matching criteria* | (a) Secure hospital record* | (a) No intelligence disturbance were seen at time of birth* | (a,b) Demographic data were provided for statistical analysis** | (a) Independent blind assessment with WISC-R and K-ABC* | (a) Yes, follow up for 10 year of age* | (c)  Participation rate  151/248 (61%) ICSI  Control children from surrounding schools 37.5% response rate (n-153) | (a) ICSI vs NC*  (b) Singleton only* | 7(2) |
| Ludwig et al. (2009)a | (b) Children born in multiple centers in Lubeck, Kiel, Hamburg, Magdeburg, berlin, Bremen, hannover, Essen, Munster Germany* | (a) Naturally conceived children from the same area and inclusion criteria* | (a) Data sets from previous study and government record* | (a) Intelligence quotient were not assessed at time assessment* | (a,b) Family background and other cofounders were provided for statistical analysis** | (a) Independent blind assessment with  Zimmer/Volkamer motor test MOT for motoric examination  + K-ABC for intelligence and abilities  + Interview for medical history  + Questionnaire* | (a) Yes, follow up for children at 4-6 year of age* | (b) Complete test results were available for 271 ICSI children and 268 control children* | (a) ICSI vs NC*  (b) Singleton only* | 10(2) |
| Mains et al. (2010)a | (c) Children born in University of Iowa Hospitals and clinics in Iowa, Canada | (c) No clear description and data available | (a) Secure hospital record* | (a) Score were not available at time of enrollment* | (a,b) Family background and other cofounders were adjusted for statistical analysis**  No data available for further analysis | (c) Self-reported ITBS, ITED score | (a) Yes, follow up at 8-17 years of age* | (b) TBS/ITED scores at third grade or higher were available on 423 (91.4%) IVF children (308 IVF cycles, 272 women) enrolled in the study* | (a) ICSI, IVF*  (b) Singleton, twins, multiple*  (c) Fresh, frozen* | 6(3) |
| [Nekkebroeck et al. (2008)](about:blankNekkebroeck%20et%20al.%20(2008)) | (b) Parents of PGD, PGS and ICSI children were recruited from the register of the Centre for Medical Genetics of the UZ Brussels* | (a) controls were selected to match the initial cohort of 70 PGD/PGS cases as closely as possible for gender, maternal educational level (high:higher education qualification or a degree; medium:fully passed school matriculation; low:partially passed school matriculation or no qualification at all), mother tongue and birth order (having an older sibling or otherwise)* | (a) Secure hospital record* | (b) Of the initial cohort, 70 PGD/PGS children were actually assessed for mental and motor developments* | (a,b) Gender and other cofounder were not statistically significant between groups or no correlation to the result** | (c) Parent-reported McArthur-Bates for language development | (a) Yes, follow up were done at 2 years of age* | (c) In 25.6% of cases, only the mother filled out the questionnaire (n=33), and in 2.3% of the cases, only the father filled out the questionnaire (n=3) | (a) ICSI vs NC*  (b) Singletons only* | 7(2) |
| Noori S et al. (2012)a | (c) 151 full-term infants after ART in Royan Institute, Iran | (c) No naturally conceived control | (a) Secure hospital record* | (a) Prelinguistic behaviour were not assessed at time of birth* | (b) Only gender and maternal age were provided for statistical analysis* | (c) Self report questionnaire on Early Language Milestone Scale-2 (ELM-2) | (b) No, follow up at 9 months old | (a) complete data at assessment* | (a) ICSI vs IVF*  (b) Singleton only* | 4(2) |
| Olivennes et al. (1996)a | (c) Children born between 1986-1994 in Beclere Hospital, Clamart, France | (c) No controls | (a) Secure hospital record* | (a) Scholastic performance were not assessed at time of enrollment* | Family background and other cofounders were not adjusted for statistical analysis | (c) Self-reported questionnaire on school rank | (a) Yes, follow up were done for children at 1-9 years of age | (a) Complete data at assessment* | (a) IVF only*  (b) Singleton and twins (no distiction in data)  (c) Frozen only* | 3(2) |
| Papaligoura et al. (2012) | (c)  The experimental group included 34 infants after ICSI:  26 – singleton  8 – twins  Control 26 infants IVF  14 – singeltons  12 – twins  Control NC 29 infants  23 – singleton  6 - twins  in Greece | (a) Naturally conceived children from the same hospital with matching criteria* | (a) Secure hospital record* | (a) Cognitive development were not assessed at time of enrollment* | (a,b) Family background and other cofounders were adjusted for statistical analysis** | (a) Independet blinded assessment with Bayley test for Infants* | (a) Yes, follow up were done at 12 months of age* | (a) complete data for the assessment* | (a) IVF, ICSI, NC* | 8(1) |
| [Place and Englert (2003)](about:blankPlace%20and%20Englert%20(2003)) | (c) Children born at Erasme Hospital, Brussels, Belgium | (a) Naturally conceived children born from the same hospital with matching criteria* | (a) Secure hospital record* | (a) Cognitive development were no assessed at time of birth* | (a,b) The control groups were matched as closely as possible with the ICSI group with respect to birth date, age and sex of the child, age of the mother, social class, ethnic background, family size, and birth order of the child** | (a) Independet blind assessmet with WPPSI-R and Brunet-Lezine scale* | (a) Yes, follow up were done for children aged 9 months to 5 years* | (c) Many participants lost to follow-up at 5 years of age | (a)IVF, ICSI, NC*  (b) Singleton*  (c) Fresh only* | 7(3) |
| [Ponjaert-Kristoffersen et al. (2004)](about:blankPonjaert-Kristoffersen%20et%20al.%20(2004)) | (c) Multiple centers in  USA, Belgium, Sweden  Center for Reproductive Medicine, Vrije Universiteit Brussle, Fertility Center Scandinavia, Calandesrksa Hospital, Reproductive medicine Sahlgresnka Univeristy Hospital of Goteborg, Weil medical College, Cornell University | (a) SC children from local schools and Medical Registry data with same inclusion criteria* | (a) Secure hospital record* | (a) Cognitive, motoric, and psychological development were not assessed at time of birth* | (a,b) Family background and other cofounders were provided** | (a) Independent blind assessement with WPPSI-R, PDMS, CBCL* | (a) Follow up were done at 5 years of age* | (b) 6/266 missing data from control* | (a) ICSI vs NC*  (b) Singletons only* | 8(2) |
| [Ponjaert-Kristoffersen et al. (2005)](about:blankPonjaert-Kristoffersen%20et%20al.%20(2005)) | (c)  Children aged 4.5 to 5.5 at assessment  541 ICSI  441 IVF  549 NC in 5 centers in Belgium, Denmark, Greece, Sweden, UK | (a) SC children from local schools and Medical registry data with same inclusion criteria* | (a) Secure hospital record* | (a) Cognitive and motoric development were not assessed at time of birth* | (a,b) Family background, gestational age, gender efects and other cofounders were provided** | (a) Independent assessment with WISC-R and K-ABC* | (a) Follow-up were done at 5 years of age* | (b) 1/1368 data were missing for PIQ and 28 missing for motoric development* | (a) ICSI vs NC*  (b) Singletons only* | 8(2) |
| Spangmose et al. (2017)a | (a) Data from Danish national cohort study* | (a) Naturally conceived children data from Danish national cohort study* | (a) Secure national record* | (a) Cognitive development were not assesed at time of birth* | (a,b) Adjusted for parity of the mother, gestational age, family background** | (b) Record linkage* | (a) follow-up were done at 15-16 years* | (b) Missing data on educational test scores occurred in 6.6% of adolescents aged 15–16 years for the birth cohorts 1995–1997, where all of the children according to their age should have passed the ninth grade exam at the time of data retrieval* | All-ART treatment, singleton and twins  (b) Singletons vs twins*  (c) Fresh only* | 9(2) |
| [Sutcliffe et al. (1995)](about:blankSutcliffe%20et%20al.%20(1995)) | (c) Children born in IVF unit in St. Mary’s Hospital, Manchester, UK | (a) Naturally conceived children from the same hospital* | (a) Secure hospital record* | (a) Cognitive, mental, and motoric development were not declared at time of birth* | (a,b) Family background, gestational age, gender efects and other cofounders were provided** | (a) Independent assessment with  General medical examination  + Griffith’s scale for developmental assessment* | (a) Yes, children were aged 1-5 years at follow up* | (a) complete data at the assessment* | (a)C-IVF only*  (b) singleton vs multiple*  (c) Frozen only* | 8(3) |
| Wagenaar et al. (2008)a | (c) Children born between 1986 and 1995 in VU Medical University, Amsterdam, Netherland | (a) Naturally conceived children from the same hospital* | (a) Secure hospital record* | (a) Cognitive and school performance were not assessed at time of enrollment* | (a,b) Family background, gestational age, gender efects and other cofounders were provided** | (c) Self-reported CITO test score and school performance | (a) yes, children were at 12 years of age* | (c) Of the total group of 233 IVF and 233 control children, 101 IVF children (43%) and 92 control children (40%) did take a CITO test, the remaining children, 107 children in the IVF (46%) and 112 children in the control groups (48%) did not have a CITO score available | (a) IVF only* | 6(1) |
| Wagenaar et al. (2009)a | (c) Children aged 8-18 years born between 1986-1995 in VU university | (a) Naturally conceived children from the same hospital* | (a) Secure hospital record* | (a) Cognitive and school performance were not assessed at time of enrollment* | (a,b) Adjusted differences for parity, maternal education level, gestational age and birthweight in the univariate linear mode** | (a) Independet assessment for  Standardized test for neuropsychological evaluation  + CITO test for intelligence  + Beery, Pursuit, Purdue Pegboard, Tapping for motoric* | (a) Yes, children were at 9-18 years at assessment* | (d) no description | (a) IVF only*  (b) Singletons* | 7(2) |

**Supplement Table 2**. Newcastle-Ottawa Scale for Cohort Studies: Motoric Development

| **Name of Study (Year)** | **Selection** | | | | **Comparability** | **Outcome** | | | **Additional** | **Total Score** |
| --- | --- | --- | --- | --- | --- | --- | --- | --- | --- | --- |
|  | Representativeness of the exposed cohort  (a) truly representative of the average IVF treated subjects in the community*  (b) somewhat representative for the IVF treated subjects in the community*  (c) selected group of users  (d) no description of the derivation of the cohort | Selection of the non-exposed cohort  (a) drawn from the same community as the exposed cohort*  (b) drawn from different source  (c) no description of the derivation of the non-exposed cohort | Ascertainment of exposure  (a) secure record*  (b) structured interview*  c) written self report  (d) no description | Demonstration that outcome of interest was not present at start of study  (a) yes*  (b) no | Comparability of cohort on the basis of the design or analysis  (a) study controls for gender*  (b) study controls for any additional factors* | Assessment of outcome  (a) Independent blind assessment*  (b) Record linkage*  (c) Self-report  (d) No description | Was follow-up long enough for outcomes to occur  (a) yes (1-year minium follow-up)* (b) no | Adequacy of follow-up of cohort  (a) complete follow-up, all subjects accounted for*  (b) subjects lost to follow-up are unlikely to introduce bias – small number lost <20%  (c) follow-up rate <80% and no description of those lost  (d) no description *or unclear* | mode of conception and embryo transfer method stated?  (a) Mode of conception (ICSI, IVF, etc.) stated*  (b) ET method (fresh/frozen stated)*  (c) No description |  |
| [Agarwal et al. (2005)](about:blankAgarwal%20et%20al.%20(2005)) | (c) Selected groups from Kandang Kerbau Hospital Singapore | (a) The control group comprised of naturally conceived children born during the same period at the same hospital with matching criteria* | (a) Secure record from hospital records* | (a) Neuro - developmental delay was not present at time of birth* | (a,b) Family background and other confounders are adjusted for in the statistical analysis** | (a) Inependent blind by medical diagnosis, interview, and Bayley Scale for Infant Development (PDI score)* | (a) Yes, Follow-up was done at 2 years of age* | (b) Eighty-five ICSI and 269 naturally conceived children were eligible to be enrolled in the study. Of these, parents of nine study (10%) and eight control children (3%) declined to participate in the study. Thus, 76 ICSI children and 261 controls were assessed at two years of age* | (a) ICSI, NC*  (c) Twins and singletons* | 8 (2) |
| [Balayla et al. (2017)](about:blankBalayla%20et%20al.%20(2017)) | (b) Data from the 3D-study (Decouvrir, developper, Devenir) that recruited women in first trimester in 9 sites in Quebec, Canada* | (a) Non-ART children data from 3D study with matching criteria* | (a) Secure record* | (a) Neurological outcomes were not tested at time of birth* | (a,b) Adjusted for maternal age, paternal age, maternal education, income, ethnicity, marital status, smoking intake, alcohol consumption during pregnancy, history of depression, antidepressant use, and folic acid intake** | (a) Independent medical diagnosis Bayley Scales of Infants and Toddlers at 2 years of age (PDI score)* | (a) Yes, follow up until 2 years of age* | (c)  175 children in ART (62.9%)  1345 – NC group (64.4%)  Underwent neurodevelopmental assessment at 24 months postpartum* | (b) Singleton only  Fertility treatment: IVF, ICSI, etc. | 8 (1) |
| Bowen et al. (1998)a | (c) Children born at North Shore Hospital, Sydney, Australia | (a) Naturally conceived children born from the same hospital* | (a) Hospital secure record* | (a) Cognitive and motoric assessment were not tested at time of birth* | (a) Inclusion of demographic variables as covariates in analysis of variance* | (a) Independent assessment using Bayley Scale II* | (a) Yes, follow up were done at 1 year of age* | (a) Complete data during assessment* | (a) IVF vs ICSI vs NC*  Singletons and twins no subgroups  Fresh and frozen no subgroups | 7(1) |
| [Gibson et al. (1998)](about:blankGibson%20et%20al.%20(1998)) | (c) Children conceived at Royal North Shore Hospital IVF Clinic | (a) Children conceived from the same hospital with same inclussion criteria* | (a) Secure hospital record* | (a) Intelligence disturbance were not seen at time of birth* | (a,b) Gender and other cofounders were not statistically different** | (a) Independent assessement with  + Bayley scales of infant development (PDI score)  * | (a) Yes, infants were assessed at 1 year post partum* | (b) IVF 93% (n=65)Control 98% (n=62)* | (c) Singleton only* | 8 (1) |
| [Jongbloed-Pereboom et al. (2011)](about:blankJongbloed-Pereboom%20et%20al.%20(2011)) | (c) Children conceived at University Medical Center Groningen, Netherlands | (b) Naturally conceived children from different clinics | (a) Secure hospital record* | (a) Motoric outcomes were not assessed at time of birth* | (a,b) Mean differences adjusted for maternal education, sex, gestational age, vanishing twins, birth weight, and time to pregnancy** | (a) Independent assessment with  Bayley scales of infant development II (PDI score)* | (a) yes, children were at 2 years of age at time of study* | (a) Only 1 missing data from PDI scale* | (a) IVF ICSI*  (b) Singletons*  (c) Fresh only* | 7(3) |
| Kuivurora et al. (2003)a | (a) Selected IVF children from Finnish medical birth register* | (a) Naturally conceived children drawn from the same community* | (a) Secure national record* | (a) Motoric outcomes were not assessed at time of enrollment* | No description about gender  (b) Family background, maternal age, and other cofounders were matched in the design* | (b) Record linkage* | (a) Yes, children were at 1month to 3 years of age at follow-up* | (d) No description | (a) IVF vs NC*  (b) Singleton and twins* | 7(2) |
| [Leunens et al. (2006)](about:blankLeunens%20et%20al.%20(2006)) | (c) children conceived in Academic Hospital of Vrije Universiteit Brussel, Belgium | (a) Naturally conceived children from local schools with matching criteria* | (a) Hospital secure record* | (a) Motoric outcome were not assessed at time of enrollment (birth)* | (a) Gender data were not shown, there is a gender effect on motor assessment based on statistical analysis*  (b) Family background were provided for statistical analysis* | (a) Independent blind assessment with Movement Assessment Battery for children* | (a) yes, follow up were done for 8 year old children* | (d) No description | (a) ICSI vs NC*  (b) Singleton only* | 7(2) |
| [Leunens et al. (2008)](about:blankLeunens%20et%20al.%20(2008)) | (c) Children from previous study at age 10 years | (a) Naturally conceived children from local schools with matching criteria* | (a) Secure hospital record* | (a) No intelligence disturbance were seen at time of birth* | (a)  Gender data were not shown, there is a gender effect on motor assessment based on statistical analysis*  (b) Family background were provided for statistical analysis* | (a) Independent blind assessment with K-ABC* | (a) Yes, follow up for 10 year of age* | (c)  Participation rate  151/248 (61%) ICSI  Control children from surrounding schools 37.5% response rate (n-153) | (a) ICSI vs NC*  (b) Singleton only* | 7(2) |
| Ludwig et al. (2009)a | (b) Children born in multiple centers in Lubeck, Kiel, Hamburg, Magdeburg, berlin, Bremen, hannover, Essen, Munster Germany* | (a) Naturally conceived children from the same area and inclusion criteria* | (a) Data sets from previous study and government record* | (a) Motoric outcome were not assessed at time assessment* | Gender distribution were not provided  (b) Family background and other cofounders were provided for statistical analysis* | (a) Independent blind assessment with  Zimmer/Volkamer motor test MOT for motoric examination* | (a) Yes, follow up for children at 5.5 year of age* | (b) Complete test results were available for 271 ICSI children and 268 control children* | (a) ICSI vs NC*  (b) Singleton only* | 8(2) |
| Papaligoura et al. (2012) | (c)  The experimental group included 34 infants after ICSI:  26 – singleton  8 – twins  Control 26 infants IVF  14 – singeltons  12 – twins  Control NC 29 infants  23 – singleton  6 - twins  in Greece | (a) Naturally conceived children from the same hospital with matching criteria* | (a) Secure hospital record* | (a) Cognitive development were not assessed at time of enrollment* | (a)  (b) Family background and other cofounders were adjusted for statistical analysis** | (a) Independet blinded assessment with Bayley test for Infants (PDI score)* | (a) Yes, follow up were done at 12 months of age | (a) complete data for the assessment* | (a) IVF, ICSI, NC* | 7(1) |
| [Ponjaert-Kristoffersen et al. (2004)](about:blankPonjaert-Kristoffersen%20et%20al.%20(2004)) | (c) Multiple centers in  USA, Belgium, Sweden  Center for Reproductive Medicine, Vrije Universiteit Brussle, Fertility Center Scandinavia, Calandesrksa Hospital, Reproductive medicine Sahlgresnka Univeristy Hospital of Goteborg, Weil medical College, Cornell University | (a) SC children from local schools and Medical Registry data with same inclusion criteria* | (a) Secure hospital record* | (a) Cognitive, motoric, and psychological development were not assessed at time of birth* | (a,b) Family background and other cofounders were provided** | (a) Independent blind assessement with Peabody Motor Development Scale* | (a) Follow up were done at 5 years of age* | (b) 6/266 missing data from control* | (a) ICSI vs NC*  (b) Singletons only* | 8(2) |
| [Ponjaert-Kristoffersen et al. (2005)](about:blankPonjaert-Kristoffersen%20et%20al.%20(2005)) | (c)  Children aged 4.5 to 5.5 at assessment  541 ICSI  441 IVF  549 NC in 5 centers in Belgium, Denmark, Greece, Sweden, UK | (a) SC children from local schools and Medical registry data with same inclusion criteria* | (a) Secure hospital record* | (a) Cognitive and motoric development were not assessed at time of birth* | (a,b) Family background, gestational age, gender efects and other cofounders were provided** | (a) Independent assessment with K-ABC* | (a) Follow-up were done at 5 years of age* | (b) 1/1368 data were missing for PIQ and 28 missing for motoric development* | (a) ICSI vs NC*  (b) Singletons only* | 8(2) |
| [Sutcliffe et al. (1995)](about:blankSutcliffe%20et%20al.%20(1995)) | (c) Children born in IVF unit in St. Mary’s Hospital, Manchester, UK | (a) Naturally conceived children from the same hospital* | (a) Secure hospital record* | (a) Cognitive, mental, and motoric development were not declared at time of birth* | Data per gender were not reported however sex ratio were reported  (b) Family background, gestational age, gender efects and other cofounders were provided** | (a) Independent assessment with  General medical examination  + Griffith’s scale for developmental assessment (locomotor score)* | (a) Yes, children were aged 1-5 years at follow up* | (a) complete data at the assessment* | (a)C-IVF only*  (b) singleton vs multiple*  (c) Frozen only* | 7(3) |

**Supplement Table 3.** Newcastle-Ottawa Scale of Cohort Studies: Behavioral and Social Development

| **Name of Study (Year)** | **Selection** | | | | **Comparability** | **Outcome** | | | **Additional** | **Total Score** |
| --- | --- | --- | --- | --- | --- | --- | --- | --- | --- | --- |
|  | Representativeness of the exposed cohort  (a) truly representative of the average IVF treated subjects in the community*  (b) somewhat representative for the IVF treated subjects in the community*  (c) selected group of users  (d) no description of the derivation of the cohort | Selection of the non-exposed cohort  (a) drawn from the same community as the exposed cohort*  (b) drawn from different source  (c) no description of the derivation of the non-exposed cohort | Ascertainment of exposure  (a) secure record*  (b) structured interview*  c) written self report  (d) no description | Demonstration that outcome of interest was not present at start of study  (a) yes*  (b) no | Comparability of cohort on the basis of the design or analysis  (a) study controls for gender*  (b) study controls for any additional factors* | Assessment of outcome  (a) Independent blind assessment*  (b) Record linkage*  (c) Self-report  (d) No description | Was follow-up long enough for outcomes to occur  (a) yes (1-year minium follow-up)* (b) no | Adequacy of follow-up of cohort  (a) complete follow-up, all subjects accounted for*  (b) subjects lost to follow-up are unlikely to introduce bias – small number lost <20%  (c) follow-up rate <80% and no description of those lost  (d) no description *or unclear* | mode of conception and embryo transfer method stated?  (a) Mode of conception (ICSI, IVF, etc.) stated*  (b) ET method (fresh/frozen stated)*  (c) No description |  |
| [Agarwal et al. (2005)](about:blankAgarwal%20et%20al.%20(2005)) | (c) Selected groups from Kandang Kerbau Hospital Singapore | (a) The control group comprised of naturally conceived children born during the same period at the same hospital with matching criteria* | (a) Secure record from hospital records* | (a) Neuro - developmental delay was not present at time of birth* | (a,b) Family background and other confounders are adjusted for in the statistical analysis** | (c) Self-reported Vineland score for infant behavior | (a) Yes, Follow-up was done at 2 years of age* | (b) Eighty-five ICSI and 269 naturally conceived children were eligible to be enrolled in the study. Of these, parents of nine study (10%) and eight control children (3%) declined to participate in the study. Thus, 76 ICSI children and 261 controls were assessed at two years of age* | (a) ICSI, NC*  (c) Twins and singletons* | 7 (2) |
| [Barnes et al. (2004)](about:blankBarnes%20et%20al.%20(2004)) | (b) Five centers from Belgium, Denmark,, Greece, Sweden, UK* | (a) Control of naturally conceived children from local schools with maternal age, maternal education and parental socio-economic status, gender and birth order* | (a) National birth register from each country except UK and Belgium* | (a) Behavioral problems were not seen at time of birth* | NC controls were selected according to the above criteria and the groups were matched as closely as possible in each country to the ICSI and IVF groups for maternal age, maternal education and parental socio-economic status, gender and birth order** | (c) Self reported  + Child behavior checklist  + general Health Questionnaire  + Parental Stress Index  + Dyadic Adjustment Scale  + Parental Acceptance-Rejection Questionnaire  + McDevitt and Carey questionnaire | (a) yes, follow-up at 5 years of age* | (c) CBCL score; Mother (ICSI= 345/484, IVF = 301/403, NC = 310/502) Father (ICSI = 193, IVF+ 191, NC=175) | (a) IVF, ICSI,NC*  (b) Singleton only* | 7(2) |
| [Bay et al. (2014)](about:blankBay%20et%20al.%20(2014)) | (a) A cohort of 1782 children sampled from the Danish National Birth Cohort* | (a) Children born from fertile parents from the same population* | (b) Structrued interview* | (a) Behavioral outcome were not assessed before study* | (a,b) Adjusted for maternal age, intelligence, parental educational level, parity, smoking in pregnancy, average alcohol consumption in pregnancy, maternal body mass index, child gender, age at testing, and testing psychologist** | (c) Self-reported Teach, BRIEF questionnaire* | (a) Yes, follow up were done at 5 years of age* | (c) Overall number of participants. Due to complete case analyses, the number of participants for the individually outcomes was: Full IQ (n=1729), Verbal IQ (n=1730), Performance IQ (n=1730), Overall attention score (n=1477), Sustained attention score (n=1570), Selective attention score (n=1595), General Executive Composite (parent version:n=1727, teacher version:n=1507), Behavioural Regulation Index (parent version: n=1727, teacher version:n=1512), Metacognition index (parent version:n=1727, teacher version:n=1507) | (b) Singleton only | 7(1) |
| Cederblad et al. (1996)a | (c) Data from children conceived in University Hospital of Lund and Malmo, Sweden | (a) Non-IVF chidlren born from the same hospital* | (a) Secure hospital record* | (a) Behavioral outcomes were not assessed at the beginning of study* | (a) Separate subgroup for gender* | (c) Self-reported Achenbach CBCL and  (b)Semi-structured interview on child's current behavior* | (a) Yes, children assessed were at 33-85 months of age* | (d) No description | (a) IVF only* | 7(1) |
| [Gibson et al. (1998)](about:blankGibson%20et%20al.%20(1998)) | (c) Children conceived at Royal North Shore Hospital IVF Clinic | (a) Children conceived from the same hospital with same inclussion criteria* | (a) Secure hospital record* | (a) Behavioral outcome were not assessed at the start of study* | (a,b) Gender and other cofounders were not statistically different** | (c) Self-reported CBCL and TRF score | (a) Yes, infants were assessed at 1 year post partum* | (b) IVF 93% (n=65)Control 98% (n=62)* | (c) Singleton only* | 7(1) |
| [Jongbloed-Pereboom et al. (2011)](about:blankJongbloed-Pereboom%20et%20al.%20(2011)) | (c) Children conceived at University Medical Center Groningen, Netherlands | (a) Naturally conceived children from different clinics with matching criteria* | (a) Secure hospital record* | (a) Behavioral outcome were not assessed at the start of study* | (a,b) Mean differences adjusted for maternal education, sex, gestational age, vanishing twins, birth weight, and time to pregnancy** | (c) Self-reported Achenbach Child Behavior Checklist (CBCL) | (a) yes, children were at 2 years of age at time of study* | (a) Only 1 missing data from PDI scale* | (a) IVF ICSI*  (b) Singletons*  (c) Fresh only* | 7(3) |
| McMahon et al. (2013)a | (b) Seven hospital clinics and private hospital in 2 big cities in Australia* | (a) Spontaneous conceived children derived from the same hospital* | (a) Secure hospital record* | (a) Behavioral outcome were not assesed at start of study* | (b) Interaction terms could be specified as follows maternal age ×trait anxiety; maternal age×state anxiety; maternal age×pregnancy-specific anxiety; mode of conception×trait anxiety, mode of conception×state anxiety; mode of conception×pregnancy-specific anxiety* | (a) Short temperament scale for infants* | (b) No, children were at 4 months post-partum | (a) Some missing data* | (d) No description | 7 |
| Montgomery et al. (1999)a | (c) children born at Jones Institute | (b) Drawn randomly from national data | (a) secure hospital record* | (a) Behavioral outcome were not assessed at the start of study* | (a) Different data subset for gender were provided* | (c) Self-questionnare  Achenbach Child Behavior Checklist 4-18 years, Youth self-report questionnaire | (a) yes, children were over 4 years of age* | (a) Complete questionnaire during the study* | (a) IVF only*  (b) Singleton and multiple, no statistical differences between singletons and twins | 5(1) |
| Nakajo et al. (2004)a | (c) Children born at Kyono Reproduction research Center and Ladies Clinic, Miyagi, Japan | (a) Naturally conceived chidlren born from the same hospital* | (a)Secure hospital record* | (a) Motoric outcomes were not assessed at time of birth* | Gender and other confounders are not matched in the design nor adjusted for in the statistical analysis (not provided) for motoric outcome | (c) Questionnaire from Japanese Ministry of Health and Welfare for physical growth and mental development* | (a) Yes, children were 1 month to 3 years of age* | (a) Complete questionnare from the participants* | C-IVF and ICSI vs NC no distiction in data  (b) Singleton, multiple, twins in different subset data*  FET vs fresh no distiction in behavioral data | 6(1) |
| [Nekkebroeck et al. (2008)](about:blankNekkebroeck%20et%20al.%20(2008)) | (b) Parents of PGD, PGS and ICSI children were recruited from the register of the Centre for Medical Genetics of the UZ Brussels* | (a) controls were selected to match the initial cohort of 70 PGD/PGS cases as closely as possible for gender, maternal educational level (high:higher education qualification or a degree; medium:fully passed school matriculation; low:partially passed school matriculation or no qualification at all), mother tongue and birth order (having an older sibling or otherwise)* | (a) Secure hospital record* | (b) Of the initial cohort, 70 PGD/PGS children were actually assessed for mental and motor developments* | (a,b) Gender and other cofounder were not statistically significant between groups or no correlation to the result** | (c) Parent-reported Short Temperament Scale for Toddler and CBCL for behavior | (a) Yes, follow up were done at 2 years of age* | (c) In 25.6% of cases, only the mother filled out the questionnaire (n=33), and in 2.3% of the cases, only the father filled out the questionnaire (n=3) | (a) ICSI vs NC*  (b) Singletons only* | 7(2) |
| [Ponjaert-Kristoffersen et al. (2004)](about:blankPonjaert-Kristoffersen%20et%20al.%20(2004)) | (c)  Children aged 4.5 to 5.5 at assessment  541 ICSI  441 IVF  549 NC in 5 centers in Belgium, Denmark, Greece, Sweden, UK | (a) SC children from local schools and Medical registry data with same inclusion criteria* | (a) Secure hospital record* | (a) Behavioral development were not assessed at time of birth* | (a,b) Main effect (conception mode) for three-way ANOVA (conception mode£medical centre£gender); (conception mode£educational level £age of the mother at birth** | (c) Self-reported CBCL and Parenting stress inventory | (a) Follow-up were done at 5 years of age* | (b) 1/1368 data were missing for PIQ and 28 missing for motoric development* | (a) ICSI vs NC*  (b) Singletons only* | 7(2) |
| [Punamaki et al. (2016)](about:blankPunamaki%20et%20al.%20(2016)) | (a)  Couples who conceived by ART in finnish fertility clinics from 18-20 weeks gestation (TI n = 860), and 2 moths (T2= 711), an 12 months (T3 = 587) post partum.  The families participated again when the children were 7-8 years old (T4, n=533)  At T4, 255 ART families and 278 NC families participated* | (a) naturally conceived children from the same community* | (a) Secure national record* | (a) Mental health problems were not seen at birth* | (a,b) Adjusted for gender and other cofounders were presented for statistical analysis** | (a)  Parent Rating Scales (PRS-C) of the Behavioral Assessment System for Children (BASC) for mental health problems  + Assertion-dimension of parent version of the Social Skills Rating System (SSRS) for chil’s social developmental problems  + Child’s cognitive developmental problems by Five to Fifteen (FTF)* | (a) Yes, children were 7-8 years of age at assessment* | (c) Mother reported data 510/533, father reported data 310/533 | (a) IVF vs ICSI*  (b) Singletons only* | 7(2) |
| [Sutcliffe et al. (1995)](about:blankSutcliffe%20et%20al.%20(1995)) | (c) Children born in IVF unit in St. Mary’s Hospital, Manchester, UK | (a) Naturally conceived children from the same hospital* | (a) Secure hospital record* | (a) Cognitive, mental, and motoric development were not declared at time of birth* | Data per gender were not reported however sex ratio were reported  (b) Family background, gestational age, gender efects and other cofounders were provided** | (a) Independent assessment with  General medical examination  + Griffith’s scale for developmental assessment (personal-social index)* | (a) Yes, children were aged 1-5 years at follow up* | (a) complete data at the assessment* | (a)C-IVF only*  (b) singleton vs multiple*  (c) Frozen only* | 7(3) |
| Sydsjo et al. (2002)a | (c) Toddlers born in Reproductive center at Linkoping Univeristy Hospital, Sweden | (a) Naturally conceived children from the same hospital* | (a) Secure hospital record* | (a) Toddler behavior were not assessed at time of birth* | Gender and other confounders are not matched in the design nor adjusted for in the statistical analysis (not provided) for motoric outcome | (a) Toddler behavior questionnaire* | (b) No, assessement were done at 11-15 month toddler | (d) No description | (d) no description | 4 |
| [Wagenaar et al. (2011)](about:blankWagenaar%20et%20al.%20(2011)) | (c) Children born in VU University Medical center | (a) Naturally conceived chidlren from the same hospital* | (a) Secure hospital record* | (a) Behavior were not assessed at time of birth* | (a) Gender data were provided for statistical analysis*  (b) Age of the child at assessment, gestational age, parity, and parental highest education level in the multiple linear mode* | (c) Youth self-Report questionnaire | (a) Yes, children were aged 11-18 years at assessment* | (a) complete data for the questionnaire* | (a) IVF vs NC*  (b) Singletons* | 7(2) |
| [Wagenaar et al. (2009)b](about:blankWagenaar%20et%20al.%20(2009)b) | (c) Children born in VU University Medical center | (a) naturally conceived children born in the same hospital* | (a) secure hospital record* | (a) Behavior were not assessed at time of birth* | (a) Gender data were provided for statistical analysis*  (b) Age of the child at assessment, gestational age, parity, and parental highest education level in the multiple linear mode* | (a) CBCL and TRF* | (a) Yes, children were aged 9-18 years of age* | (c) Missing data from the parents and teacher reported questionnaire | (a) IVF vs NC*  (b) Singletons* | 7(2) |

**Supplement Table 4.** Newcastle Ottawa Scale for the Case-Control Studies

| **Name of Study (Year)** | **Selection** | | | | **Comparability** | **Exposure** | | | **Additional** | **Total Score** |
| --- | --- | --- | --- | --- | --- | --- | --- | --- | --- | --- |
|  | Is the case definition adequate?  (a) Yes, with independent validation* (b) Yes, (e.g. record linkage or based on self reports) (c) No description | Representativeness of the cases  (a) Consecutive or obviously representative series of cases* (b) Potential for selection biases or not stated | Selection of Controls  (a) Community controls* (b) Hospital controls (c) No description | Definition of Controls  (a) No history of disease* (end-point) (b) No description of source | Comparability of the cases and controls on the basis of the design or analysis  (a) Study controls formaternal age* (b) Study controls for any additional factors* | Ascertainment of exposure  (a) Secure record (e.g. surgical record)* (b) Structured interview blind to case/control (c) Interview not blinded to case/control (d) Written self report or medical record only (e) No description | Same method of ascertainment (a) Yes* (b) No | Non-response rate (a) Same rate for both groups* (b) Non-respondents described (c) Rate different and no designation | mode of conception and embryo transfer method stated?  (a) Mode of conception (ICSI, IVF, etc.) stated*  (b) Singleton vs twins  (c) ET method (fresh/frozen stated)*  (c) No description |  |
| [Sutcliffe et al. (2003)](about:blankSutcliffe%20et%20al.%20(2003)) | (a) Cases identified through examination* | (a) All eligible cases over a defined time/catchment area* | (a) Control (naturally conceived children) derived from the same community* | (a) Naturally conceived chidlren* | (a,b) To compare the data between groups and allow for other possible confounders, multivariate analyses were used: stepwise regression for continuous dependent variables or logistic regression for binary dependent variables** | (a) Secure hospital record* | (a) yes* | a) Same rate of both groups* | (a) ICSI vs Naturally conceived*  (b) Singletons* | 9(2) |
| [Winter et al. (2014)](about:blankWinter%20et%20al.%20(2014)) | (a) Cases identified through examination* | (a) All eligible cases over a defined time/cacthment area* | (a) Control (ICSI and SC) derived from the same community* | (a) naturally conceived and ICSI children* | (a,b) Matched as closely as possible for gender, age, birth order, maternal educational level (low: partially completed school education or no qualifications at all, medium: school education completed, high: higher education qualification or a degree)** | (a) Secure hospital record* | (a) yes* | (a) Same rate of both groups* | (a) ICSI vs NC*  (b) Singletons* | 9(2) |

**Supplement Table 5.** Characteristic of the Included Studies

| **Author (year)** | **Country/ birth range** | **Population number** | **Children**  **age at assessment** | **Inclusion Criteria** | **Exclusion Criteria** | **Outcome** | **Method of Assessment** | **Subgroup** | **Author's conclusion** | **NOS** |
| --- | --- | --- | --- | --- | --- | --- | --- | --- | --- | --- |
| [Agarwal et al. (2005)](about:blankAgarwal%20et%20al.%20(2005)) | Singapore/  1998-1999 | Singleton:  ICSI = 41  Control = 147  Multiple  ICSI = 35  Control 114 | 2 years | Children born between 1998-1999 in Kandang kerbau Hospital, Singapore | Not stated | + Mental development  + Motoric development  + Adaptive behavior (communication, social skill, daily living skill, motoric skill) | + Bayley Scale II (MDI scale)  + Bayley Scale II (PDI scale) + Vineland adaptive behaviour score | + ICSI  + Control | Children born by ICSI pregnancies did not have an adverse neurodevelopmental outcome | 8 |
| [Balayla et al. (2017)](about:blankBalayla%20et%20al.%20(2017)) | Canada/ 2010-2012 | Singleton:  ART (IO, IUSI, IVF, ICSI) = 175  Non-ART = 1345 | 24 months | The 3D-Study enrolled: 1) pregnant women between 8 0/7 and 13 6/7 completed weeks of gestation and 2) planning delivery in a 3D-Study–associated hospital | 1) women younger than 18 years of age, 2) illegal intravenous drug users, 3) nonEnglish or French speakers, 4) severe illnesses or life-threatening conditions, and 5) multiple pregnancies, which includes twins or higher order multiples and mothers whose previous pregnancies had been enrolled in the study | + Mental development  + Motoric development  + Language development | +Bayley Scales of Infant and Toddler Development, 3rd Edition:  - cognitive composite  - motor composite  - gross motor  - fine motor  +MacArthur-Bates Communicative Development Inventories Scale Scores | No subgroup | Children born after ART had similar cognitive, motor, and language development as children born after natural conception at 2 years of age | 8 |
| [Bay et al. (2014)](about:blankBay%20et%20al.%20(2014)) | Denmark/ 2003-2008 | Singleton:  3478 singletons from the DNBC was invited to the LDPS and 1782 (51.2%) participated in the neuropsychological assessments | 5 years | This follow-up study is based on the mother–child pairs participating in the Lifestyle During Pregnancy Study (LDPS), a study investigating the influence of different prenatal exposures on the child’s neurodevelopment at age five | The only exclusion criterion was the inability to speak Danish | + Intelligence (Full scale IQ, verbal IQ, performance IQ)  + Attention (sustained, selective)  + Executive function(General, behavioural, metacognition inex) | + Weschler Preschool and Primary Scale of Intelligence-Revised (Full score IQ, performance IQ, verbal IQ)  + Test of Everyday Attention for Children at Five (sustanained attention, selective attention)  +Behavior Rating Inventory of Executive Functions (parents and teacher version): General executive composite, behavioral regulation index, metacognition index | + Fertile parents  + Subfertile parents  + Fertility treated | This study suggests that parental subfertility and fertility treatment are unrelated to offspring intelligence, attention and executive functions | 8 |
| [Gibson et al. (1998)](about:blankGibson%20et%20al.%20(1998)) | Australia/  1992-1995 | 65 singleton infants conceived through in-vitro fertilization (IVF) and 63 matched controls | 4 months to 12 months | primiparous, singleton pregnancy, 28 years or older, living with the father of the child, and adequate English language skills to complete selfreport measures and interviews | Not stated | + mental and psychomotor development  + Language development  + Social development  + Behavioural measure (data not shown)  + Temperament (data not shown) | + Bayley Scale of infant development 2nd edition (MDI and PDI scale)  + Receptive–Expressive Emergent Language Test, 2nd edition (Receptive language EQ, expressive language EQ)  + Vineland Adaptive behavior (social quotient)  + Bayley Behaviour Rating Scale  + Behavior Checklist  + Short Temperament Scale for Toddlers | + IVF group  + Control group | Overall, singleton children conceived through IVF demonstrate appropriate general development at 1 year of age. The higher reported behaviour difficulty experienced by IVF mothers may reflect their concerns about the well-being and adjustment of their child during the first year. | 8 |
| [Heineman et al. (2019)](about:blankHeineman%20et%20al.%20(2019)) | Netherlands/  2005-2006 | Singletons born following IVF or ICSI with OS (n=57), born after modified natural cycle IVF/ICSI (MNC-IVF/ICSI;n=46) and born after natural conception to subfertile couples (Sub-NC;n=66) | 9 years | Not stated | Couples treated with cryopreserved or donated oocytes or embryos were not suitable for inclusion | + Intelligence quotient  + Attention and executive functions; memory and learning; and social cognition  + Behavioral problem | + Weschler Adult Intelligence Scale-III (full IQ, performance IQ, verbal IQ)  + NEPSY-II (domain attention, domain social, domain memory and executive function)  + CBCL and TRF (total behavior problem, internalizing, externalizing) | + OS/ IVF-ICSI  + MNC/ IVF-ICSI  + Sub-NC | Our study indicated that OS and thein vitro laboratory procedures or the combination of both and TTP were not associated with cognitive and behavioural outcome at 9 years | 8 |
| [Jongbloed-Pereboom et al. (2011)](about:blankJongbloed-Pereboom%20et%20al.%20(2011)) | Netherland/ 2005-2006 | Singletons born after controlled ovarian hyperstimulation (COH)–IVF (n¼66) and modified natural cycle–IVF (n¼56), singletons born to subfertile couples who conceived naturally (subfertile–naturally conceived, n¼87), and a reference group of 101 2-year-old singletons born to fertile couples | 2 year | Not stated | Excluded were children born after cryopreservation or donation of oocytes or embryos for ART groups and any form of assisted conception and a time to pregnancy of>1 year for control group | + Mental and Psychomotor development  + Behavior | + Bayley Scale of Infant Development II (MDI and PDI score)  + child behavior checklist: internalising, externalising, emotionally reactive, anxious/depressed, somatic complaints, withdrawn, sleep problem, attention problem, aggresive behavior) | + COH-IVF  + MNC-IVF  + Sub-NC  + Subfertile  + Control NC | This present relatively small study found no differences in cognitive and psychomotor development and behavior at 2 years in children born after COH-IVF or modified natural cycle–IVF or naturally conceived children of subfertile parents. Replication of the study is needed before firm conclusions can be drawn. Furthermore, long-term follow-up is needed to confirm these findings in older children | 8 |
| [Leunens et al. (2006)](about:blankLeunens%20et%20al.%20(2006)) | Belgium/ 1993-1995 | 151 8-year-old singletons born through ICSI after 32 weeks of gestation were compared with those of 153 singletons ofthe same age born after spontaneous conception (SC) | 8 years | The children’s inclusion criteria were being born after 32 weeks of gestation, singleton and native language Dutch withat least one European parent | multiple births, linguistic barriers and sociocultural differences | + Intelligence  + Motoric development | + Weschler Intelligence Scale for Children-Revised (Verbal IQ with subtests and performance IQ)  + Movement ABC (total motor, manual skill, ball skill, balance) | + ICSI  + NC | In this follow-up study, ICSI and SC children show a comparable cognitive and motor development until the age of 8 years | 7 |
| [Leunens et al. (2008)](about:blankLeunens%20et%20al.%20(2008)) | Belgium/  1993-1995 | Singletons born after ICSI (n=109) and controls born after spontaneous conception (SC) (n=90) | 10 years | The children’s initial inclusion criteria were: being born after 32 weeks of gestation, singleton and native language Dutch, with at least one European parent | multiple births, linguistic barriers and socio-cultural differences | + Intelligence  + Motoric development | + Weschler Intelligence Scale Revised (full scale IQ, verbal IQ with subtest and performance IQ with subtest)  + Movement ABC (total motor skill, maula skill, ball skill, balance) | + ICSI  + NC | In this follow-up study, ICSI and SC children show a comparable cognitive and motor development until the age of 10. These findings are in line with those obtained at age 8. | 7 |
| [Nekkebroeck et al. (2008)](about:blankNekkebroeck%20et%20al.%20(2008)) | Belgium/ 2002-2005 | 29 children, of whom 41 were conceived after PGD/PGS, 35 after ICSI and 53 naturally | 2 years | Understand Dutch | Twins, very low birthweight, APGAR score less than 9 after 10 min | + behavioral problems  + Temperament  + Language development | + CBCL (total problem, internalizing, externalizing problems)  + Short Temperament Scale for Toddlers (easy/average/ difficult temperament, approach, cooperation, persistence, rhytmicity, distractibility, reactivity)  + McArthur Communicative Inventories | + PGD/PGS  + ICSI  + NC | PGD/PGS conception does not adversely affect children’s socio-emotional and language development at age 2, nor did parents differ from ICSI and NC parents for parental stress and health status | 7 |
| [Place and Englert (2003)](about:blankPlace%20and%20Englert%20(2003)) | Belgium/ 1998-2000 | 66 ICSI-conceived children prospectively compared with 52 IVF-conceived and 59 spontaneously conceived children | 9 months, 18 months, 3 years, 5 years | All children included in the study were full-term singletons so as to test the possible influence of the ICSI procedure without having the interference of other difficulties associated with multiple births and prematurity | pregnancies obtained after frozen and thawed ETs (either IVF or ICSI) as well as children with a birth weight of2,500 g were excluded from the study | + Development tal outcome  + Intelligence | + Brunet-Lezinne scale for 9 and 18 months children (psoture, coordination, language, sociability, developmental quotient)  + Weschler Preschool and Primary Scale for Intelligence (performance IQ, verbal IQ, full IQ) | + ICSI  + IVF  + NC | This pilot study shows that throughout the preschool period, ICSI-conceived children have psychomotor and intellectual development similar to that of IVF-conceived and spontaneously conceived children. These conclusions need to be confirmed by multicenter studies | 7 |
| [Ponjaert-Kristoffersen et al. (2004)](about:blankPonjaert-Kristoffersen%20et%20al.%20(2004)) | Belgium, Germany, America/ | 300 ICSI children and 300 control children | 5 years | Not stated | Exclusion criteria were multiple birth and birth at ,32 weeks of gestational age, and maternal or child language different from the language of the country (Dutch, Swedish and English) | + Intelligence  + Motoric outcome  + Behavior problem  + Parenting stress | + Weschler Preschool and Primary School Intelligence Revised (verbal IQ and subtests, performance IQ and subtests, full scale IQ)  + Peabody development motor scale (fine and gross motor)  + Child behavior checklist (internalizing, externalizing, behavioral problems)  + Parenting Stress Index | + ICSI  + NC | Although the finding that a higher proportion of ICSI children obtained scores below the cut-off on some of the visual–spatial subscales of the WPPSI-R warrants further investigation, ICSI does not appear to affect the psychological well-being or cognitive development at age 5 | 8 |
| [Ponjaert-Kristoffersen et al. (2005)](about:blankPonjaert-Kristoffersen%20et%20al.%20(2005)) | Belgium, Denmark, Greece, Sweden, UK | 511 ICSI-conceived children were compared with 424 IVF-conceived children and 488 NC controls | 4.5-5.5 years | singleton, white, born after32 weeks of gestation, and first or second born, with a native language of English, Dutch, Danish, Swedish, or Greek, respectively. NC control subjects were selected according to the aforementioned criteria and were also matched with respect to maternal education, parental socioeconomic status, gender, and birth order | Not stated | + Intelligence  + Coordination during performace | + Wechsler Preschool and Primary Scale of IntelligenceRevised (WPPSI-R) (verbal IQ, performance IQ, total IQ with subtests)  + McCarthy scale (MCS index) | + ICSI  + IVF  + NC | This study includes a substantial number of children from several European countries. Apart from a few interaction effects between mode of conception and demographic variables, no differences were found when ICSI, IVF, and NC scores on the WPPSI-R and MSCA Motor Scale were compared. | 8 |
| [Sutcliffe et al. (1995)](about:blankSutcliffe%20et%20al.%20(1995)) | United Kingdom/ 1989-1994 | 91 children from cryopreserved embryos and 83 control children | 1-5 years | Control group: children were either siblings, cousins, or peers of the study group. | Not stated | + mental development  + Motoric development  + Socio-behaviour development | + Griffith scale (locomotor, personal-social, hearing and speech, eye-hand coordination, performance) | + Cryo  + NC | Overall, the development in children from cryopreserved embryos did not cause concern though formal testing had highlighted small differences compared with other children conceived normally and of a similar social class | 8 |
| [Sutcliffe et al. (2003)](about:blankSutcliffe%20et%20al.%20(2003)) | United Kingdom, Australia | Fifty-eight singleton children born after ICSI and 38 normally conceived singleton children (controls), matched for relevant sociodemographic characteristics, from Australia and 208 case-patients and 221 controls from the United Kingdom | 0-15 months | Not stated | Twins and triplets were excluded from the study | + mental development  + Motoric development  + Socio-behaviour development | + Griffith scale (locomotor, personal-social, hearing and speech, eye-hand coordination, performance) | + ICSI  + Control | Children conceived after ICSI did not differ from their naturally conceived peers in physical health or development at ages up to 15 months. | 9 |
| [Winter et al. (2014)](about:blankWinter%20et%20al.%20(2014)) | Belgium/ 1995-1997 | PGD (n=47), 49 ICSI, 48 SC | 5 to 6 years | Children were eligible if they were conceived naturally without any kind of medical intervention, singleton, Caucasian, Dutch speaking and born after 32 weeks of gestation | Multiples and very prematurely born children were excluded | + cognitive development  + Motor development | + Wechsler Preschool and Primary Scale of Intelligence III (Full IQ, VIQ with subtests, PIQ with subtests)  + M-ABC (Total movement, ball skill, balance skill) | + PGD  + ICSI  + NC | Long-term follow-up of children born after embryo biopsy, in this case for PGD, is needed to confirm that the development of these children remains comparable to ICSI and SC children. Our findings do support the safety of the PGD technique and will reassure patients with hereditary genetic diseases regarding the health of their future offspring conceived with PGD | 7 |
| [Barnes et al. (2004)](about:blankBarnes%20et%20al.%20(2004)) | Belgium, Denmark, UK, Greece, Sweden/ | ICSI = 228, IVF= 214, NC=209 | 5 years | singleton, Caucasian, born after at least 32 weeks gestation, firrst or second born and whose mother tongue was respectively English, Dutch, Danish, Swedish or Greek | Not stated | + Family relation test  + Parental wellbeing  + Parenting stress  + Marital relationship  + Parent-child relationship  + Children socioemotional development | + Benne - Anthony Family Relation test  + General Health Questionnaire  + Parenting Stress Index  + The Dyadic Adjustment Scale  + Parent±Child Dysfunctional Interaction' subscale (P-CDI)  + McDevit and Carrey temperament questionnaire  + CBCL  + PSI Difficult Child subscale | + ICSI  + IVF  + NC | The study con®rms the results of previous work with IVF families. This should be encouraging for families using these techniques in the future |  |
| [Punamaki et al. (2016)](about:blankPunamaki%20et%20al.%20(2016)) | Finland/ 1999 | 255 singleton ART children (IVF and ICSI) were compared with 278 NC children | 7 to 8 years | Conceived in Finnish ferility clinics, undergone routine ultrasound examination, singleton, pregnancies from own gametes | Not stated | + Mental health problems  + Social problems  + cognitive problems | + Parent rating scale of Behavioral Assessment System for Children (BASC)  + Social Skills Rating System  + Five to Fifteen (FTF) | + ART  + NC | ART children do not differ with regard to mental health or social and cognitive developmental problems when compared with controls, but some gender-specific differences do exis |  |
| [Wagenaar et al. (2009)b](about:blankWagenaar%20et%20al.%20(2009)b) | Netherlands/ 1986-1995 | 139 IVF and 143 control children | 9 to 18 years | Singleton | Not stated | + Behavioral problems | + Childern Behavior Checklist Parent version (with subtests)  + Teacher reporting form (with subtest) | + IVF  + Control | Overall, behavior, and socioemotional functioning of 9–18-year-old IVF children is normal. The reduced behavior of externalizing nature reported by the parents, and teacher ratings of more withdrawn/depressed behavior need further study |  |
| [Wagenaar et al. (2011)](about:blankWagenaar%20et%20al.%20(2011)) | Netherlands/ 1986-1995 | 86 IVF and 97 control adolescents | 11 to 18 years | Singleton | Not stated | + Behavioral problems | + Youth self-report (with subtest) | + IVF  + NC | Behavior and socioemotional functioning as reported by 11- to 18-year-old adolescents conceived by IVF were found to be normal. Previously found reduced behavior of externalizing nature and more withdrawn/ depressed behavior reported by parents and teachers could not be reproduced in this study. We found no significant influence of IVF conception on behavior in adolescence. |  |

**Supplement Table 6**. Summary of Meta-analysis

| **Outcome** | **Standardized Mean Difference [95% CI], p-value** | **Heterogeneity**  **(I^2^),**  **p-value** | **Egger’s**  **p-value** | **Number of Studies’ Data** |  |
| --- | --- | --- | --- | --- | --- |
| **TODDLER** | | | | |  |
| **Intelligence: Full Scale IQ** | | | | |  |
| **Summary** | **-0.12 [-0.30, 0.05], 0.16** | **0%, 0.94** | **0.506** | **4** | |
|  |  |  |  |  | |
| **Intelligence: Language or Verbal Intelligence Score** | | | | | |
| ART vs NC | 0.15 [-0.29, 0.59], 0.50 | 92%, 0.0005 | NE | 2 | |
| IVF vs NC | -0.27 [-0.50, -0.03], 0.02 | 17%, 0.30 | 0.189 | 3 | |
| ICSI vs NC | 0.14 [0.04, 0.24], 0.005 | 0%, 0.48 | 0.055 | 5 | |
| **Summary** | **0.02 [-0.13, 0.17], 0.76** | **71%, 0.0003** | **0.118** | **10** | |
|  |  |  |  |  | |
| **Intelligence: Non-Verbal Intelligence Score** | | | | | |
| **Summary** | **-0.12 [-0.24, -0.00], 0.047** | **10%, 0.34** | **0.703** | **4** | |
|  |  |  |  |  | |
| **Motoric: Full Motor Score** | | | | | |
| ART vs NC | -0.10 [-0.24, 0.04], 0.17 | 0%, 0.64 | NE | 2 | |
| IVF vs NC | -0.08 [-0.29, 0.13], 0.48 | 0%, 0.62 | 0.932 | 3 | |
| ICSI vs NC | -0.04 [-0.19, 0.12],0.62 | 44%, 0.13 | 0.450 | 5 | |
| **Summary** | **-0.05 [-0.13, 0.04], 0.27** | **6%, 0.38** | **0.575** | **10** | |
|  |  |  |  |  | |
| **Motoric: Gross Motor Score** | | | | | |
| IVF vs NC | 0.01 [-0.25, 0.28], 0.93 | 0%, 0.84 | NE | 2 | |
| ICSI vs NC | 0.03 [-0.23, 0.28], 0.83 | 76%, 0.006 | 0.567 | 4 | |
| **Summary** | **0.02 [-0.16, 0.21], 0.79** | **61%, 0.03** | **0.388** | **6** | |
|  |  |  |  |  | |
| **Motoric: Fine Motor Score** | | | | | |
| IVF vs NC | -0.33 [-0.60, -0.07], 0.01 | 0%, 0.78 | NE | 2 | |
| ICSI vs NC | -0.07 [-0.21, 0.06], 0.28 | 25%, 0.26 | 0.545 | 4 | |
| **Summary** | **-0.14 [-0.27, 0.00], 0.055** | **35%, 0.17** | **0.332** | **6** | |
|  |  |  |  |  | |
| **Behavior: Total Behavior Problem Score** | | | | | |
| **Summary** | **-0.13 [-0.23, -0.03], 0.01** | **0%, 0.42** | **0.112** | **4** | |
|  |  |  |  |  | |
| **Behavior: Internalizing Behavior Score** | | | | | |
| **Summary** | **-0.11 [-0.23, 0.02], 0.09** | **22%, 0.28** | **0.112** | **4** | |
|  |  |  |  |  | |
| **Behavior: Externalizing Behavior Score** | | | | | |
| **Summary** | **-0.17 [-0.27, -0.07], 0.001** | **0%, 0.65** | **0.761** | **4** | |
|  |  |  |  |  | |
| **Social Skill Score** | | | | | |
| IVF vs NC | -0.06 [-0.27, 0.15], 0.57 | 0%, 0.57 | 0.546 | 3 | |
| ICSI vs NC | -0.05 [-0.15, 0.04], 0.28 | 0%, 1.00 | 0.010 | 5 | |
| **Summary** | **-0.06 [-0.15, 0.03], 0.22** | **0%, 0.99** | **0.188** | **8** | |
|  |  |  |  |  | |
|  |  |  |  |  | |
| **PRE-SCHOOL AGE** | | | | | |
| **Intelligence : Full Scale Intelligence Quotient** | | | | | |
| ART vs NC | 0.00 [-0.28, 0.29], 0.98 | 54%, 0.11 | 0.004 | 3 | |
| IVF vs NC | -0.10 [-0.22, 0.02], 0.11 | 0%, 0.38 | 0.167 | 3 | |
| ICSI vs NC | -0.04 [-0.17, 0.09], 0.59 | 59%, 0.01 | 0.237 | 10 | |
| **Summary** | **-0.05 [-0.14, 0.05], 0.31** | **50%, 0.01** | **0.438** | **15** | |
|  |  |  |  |  | |
| **Intelligence: Verbal Intelligence Score** | | | | | |
| ART vs NC | 0.01 [-0.17, 0.20], 0.88 | 48%, 0.10 | 0.802 | 5 | |
| IVF vs NC | -0.30 [-0.66, 0.07], 0.11 | 74%, 0.02 | 0.818 | 3 | |
| ICSI vs NC | -0.10 [-0.34, 0.14], 0.40 | 85%, <0.00001 | 0.554 | 9 | |
| **Summary** | **-0.09 [-0.24, 0.05], 0.21** | **79%, <0.0001** | **0.599** | **17** | |
|  |  |  |  |  | |
| **Intelligence: Quantitative/ Arithmetic Score** | | | | | |
| **Summary** | **0.01 [-0.07, 0.09], 0.80** | **18%, 0.30** | **0.338** | **6** | |
|  |  |  |  |  | |
| **Intelligence: Non-Verbal Intelligence Score** | | | | | |
| ART vs NC | -0.06 [-0.20, 0.08], 0.39 | 6%, 0.36 | 0.139 | 4 | |
| IVF vs NC | -0.15 [-0.38, 0.08], 0.20 | 45%, 0.16 | 0.509 | 3 | |
| ICSI vs NC | -0.08 [-0.24, 0.08], 0.33 | 73%, <0.0001 | 0.279 | 10 | |
| **Summary** | **-0.07 [-0.18, 0.03], 0.15** | **61%, 0.0006** | **0.348** | **17** | |
|  |  |  |  |  | |
| **Intelligence: Fluid Intelligence Score** | | | | | |
| IVF vs NC | -0.08 [-0.34, 0.17], 0.53 | 61% 0.11 | NE | 2 | |
| ICSI vs NC | 0.02 [-0.13, 0.17], 0.78 | 64%, 0.01 | 0.913 | 7 | |
| **Summary** | **-0.00 [-0.13, 0.12], 0.94** | **66%, 0003** | **0.231** | **9** | |
|  |  |  |  |  | |
| **Intelligence: Short term Memory and Processing Speed Score** | | | | | |
| **Summary** | **-0.02 [-0.13, 0.09], 0.76** | **0%, 0.62** | **0.402** | **6** | |
|  |  |  |  |  | |
| **Intelligence: Visual Spatial Intelligence Score** | | | | | |
| ART vs NC | -0.12 [-0.25, 0.01] 0.07 | 25% 0.26 | 0.392 | 3 | |
| ICSI vs NC | -0.03 [-0.12, 0.06], 0.53 | 29%, 0.19 | 0.824 | 9 | |
| **Summary** | **-0.06 [-0.13, 0.02] 0.14** | **30%, 0.15** | **0.478** | **12** | |
|  |  |  |  |  | |
| **Intelligence: Long-term memory retrieval/ learning ability score** | | | |  | |
| **Summary** | **0.02 [-0.05, 0.09] 0.53** | **0%, 0.54** | **0.443** | **6** | |
|  |  |  |  |  | |
| **Intelligence: Executive Function** | | | | | |
| **Summary** | **-0.15 [-0.49, 0.18],0.37** | **74%, 0.010** | **0.215** | **4** | |
|  |  |  |  |  | |
| **Motoric: Total Motor** | | | | | |
| **Summary** | **-0.05 [-0.19, 0.09], 0.50** | **75%, 0.0002** | **0.399** | **8** | |
|  |  |  |  |  | |
| **Motoric: Gross Motor** | | | | | |
| **Summary** | **0.05 [-0.23, 0.33], 0.72** | **82%, 0.0002** | **0.275** | **5** | |
|  |  |  |  |  | |
| **Motoric: Fine Motor** | | | | | |
| **Summary** | **-0.17 [-0.47, 0.12], 0.25** | **84%, <0.0001** | **0.657** | **5** | |
|  |  |  |  |  | |
| **Behavior: Parents’ CBCL Total Behavior** | | | | | |
| ART vs NC | -0.12 [-0.53, 0.29], 0.56 | 50%, 0.16 | NE | 2 | |
| IVF vs NC | -0.31 [-0.70, 0.08], 0.12 | 78%, 0.01 | 0.858 | 3 | |
| ICSI vs NC | -0.10 [-0.20, 0.01], 0.08 | 0%, 0.41 | 0.114 | 3 | |
| **Summary** | **-0.15 [-0.28, -0.02], 0.02** | **50%, 0.05** | **0.715** | **8** | |
|  |  |  |  |  | |
| **Behavior: Parents’ CBCL Internalizing** | | | | | |
| ART vs NC | -0.14 [-0.70, 0.42], 0.62 | 73%, 0.05 | NE | 2 | |
| IVF vs NC | -0.11 [-0.27, 0.05], 0.18 | 10%, 0.33 | 0.892 | 3 | |
| ICSI vs NC | -0.05 [-0.16, 0.06], 0.35 | 0%, 0.91 | 0.323 | 3 | |
| **Summary** | **-0.08 [-0.16, 0.00], 0.06** | **0%, 0.44** | **0.790** | **8** | |
|  |  |  |  |  | |
| **Behavior: Parents’ CBCL Externalizing** | | | | | |
| ART vs NC | -0.12 [-0.48, 0.24], 0.52 | 36%, 0.21 | NE | 2 | |
| IVF vs NC | -0.15 [-0.29, -0.01], 0.04 | 0%, 0.76 | 0.452 | 3 | |
| ICSI vs NC | -0.12 [-0.27, 0.03], 0.12 | 32%, 0.22 | 0.181 | 4 | |
| **Summary** | **-0.14 [-0.23, -0.06], 0.001** | **0%, 0.59** | **0.111** | **9** | |
|  |  |  |  |  | |
| **Behavior: Teachers’ TRF Total Behavior** | | | | | |
| **Summary** | **-0.11 [-0.55, 0.34], 0.64** | **57%, 0.10** | **0.272** | **3** | |
|  |  |  |  |  | |
| **Behavior: Teachers’ TRF Internalizing** | | | | | |
| **Summary** | **-0.08 [-0.37, 0.22], 0.61** | **14%, 0.31** | **0.697** | **3** | |
|  |  |  |  |  | |
| **Behavior: Teachers’ TRF Externalizing** | | | | | |
| **Summary** | **-0.17 [-0.44, 0.09], 0.20** | **0%, 0.39** | **0.356** | **3** | |
|  |  |  |  |  | |
| **Behavior: Social Score** | | | | | |
| **Summary** | **0.20 [-0.02, 0.42], 0.08** | **0%, 0.61** | **0.611** | **3** | |
|  |  |  |  |  | |
| **YOUNG ADOLESCENT (8-18 YEARS)** | | | | | |
| **Intelligence: Language or Reading or Vocabulary** | | | | | |
| **Summary** | **0.20 [0.12, 0.28], <0.00001** | **94%, <0.00001** | **0.104** | **4** | |
|  |  |  |  |  | |
| **Intelligence: Mathematics** | | | | | |
| **Summary** | **0.18 [0.12, 0.25], <0.00001** | **90%, <0.00001** | **0.025** | **4** | |
|  |  |  |  |  | |
| **Behavior: Parents’ CBCL Total Behavior** | | | | | |
| **Summary** | **-0.14 [-0.36, 0.08], 0.20** | **0%, 0.58** | **NE** | **2** | |
|  |  |  |  |  | |
| **Behavior: Parents’ CBCL Internalizing** | | | | | |
| **Summary** | **-0.19 [-0.65, 0.27], 0.42** | **55%, 0.14** | **NE** | **2** | |
|  |  |  |  |  | |
| **Behavior: Parents’ CBCL Externalizing** | | | | | |
| **Summary** | **-0.18 [-0.40, 0.04], 0.11** | **0%, 0.80** | **NE** | **2** | |
|  |  |  |  |  | |
| **Behavior: Youth Self Report Total Behavior** | | | | | |
| **Summary** | **-0.07 [-0.33, 0.19],0.59** | **0%, 0.33** | **NE** | **2** | |
|  |  |  |  |  | |
| **Behavior: Youth Self Report Internalizing** | | | | | |
| **Summary** | **-0.04 [-0.39, 0.32], 0.84** | **28%, 0.24** | **NE** | **2** | |
|  |  |  |  |  | |
| **Behavior Youth Self Report Externalizing** | | | | | |
| **Summary** | **-0.03 [-0.29, 0.23], 0.81** | **0%, 0.41** | **NE** | **2** | |
